# Single cell atlas of spinal cord injury in mice reveals a pro-regenerative signature in spinocerebellar neurons

**DOI:** 10.1038/s41467-022-33184-1

**Published:** 2022-09-26

**Authors:** Kaya J. E. Matson, Daniel E. Russ, Claudia Kathe, Isabelle Hua, Dragan Maric, Yi Ding, Jonathan Krynitsky, Randall Pursley, Anupama Sathyamurthy, Jordan W. Squair, Boaz P. Levi, Gregoire Courtine, Ariel J. Levine

**Affiliations:** 1 Spinal Circuits and Plasticity Unit, National Institute of Neurological Disorders and Stroke, National Institutes of Health, Bethesda, MD USA; 2 Johns Hopkins University Department of Biology, Baltimore, MD USA; 3 Division of Cancer Epidemiology and Genetics, Data Science Research Group, National Cancer Institute, NIH, Rockville, MD USA; 4 Center for Neuroprosthetics and Brain Mind Institute, Faculty of Life Sciences, École Polytechnique Fédérale de Lausanne (EPFL), Lausanne, Switzerland; 5 NeuroRestore, Department of Clinical Neuroscience, Lausanne University Hospital (CHUV) and University of Lausanne (UNIL), Lausanne, Switzerland; 6 National Institute of Neurological Disorders and Stroke, Bethesda, MD USA; 7 Allen Institute for Brain Science, Seattle, WA USA; 8 Signal Processing and Instrumentation Section, Center for Information Technology, National Institutes of Health, Bethesda, MD USA; 9 Present Address: Centre for Neuroscience, Indian Institute of Science, Bangalore, India

**Keywords:** Molecular neuroscience, Spinal cord injury

## Abstract

After spinal cord injury, tissue distal to the lesion contains undamaged cells that could support or augment recovery. Targeting these cells requires a clearer understanding of their injury responses and capacity for repair. Here, we use single nucleus RNA sequencing to profile how each cell type in the lumbar spinal cord changes after a thoracic injury in mice. We present an atlas of these dynamic responses across dozens of cell types in the acute, subacute, and chronically injured spinal cord. Using this resource, we find rare spinal neurons that express a signature of regeneration in response to injury, including a major population that represent spinocerebellar projection neurons. We characterize these cells anatomically and observed axonal sparing, outgrowth, and remodeling in the spinal cord and cerebellum. Together, this work provides a key resource for studying cellular responses to injury and uncovers the spontaneous plasticity of spinocerebellar neurons, uncovering a potential candidate for targeted therapy.

## Abstract (web summary)

Matson et al. performed single nucleus sequencing of the “spared” spinal cord tissue distal to an injury in mice. They found that spinocerebellar neurons expressed a pro-regenerative gene signature and showed axon outgrowth after injury.

## Introduction

The brain, spinal cord, and peripheral nervous system are comprised of diverse cell types that operate together as global and local communities to enable normal physiology. Following acute trauma, a complex interplay of cellular responses shapes the outcome. Whether the tissue can restrict the damage, promote structural remodeling and functional compensation, and ultimately achieve recovery depends on a myriad of dynamic molecular changes amongst neurons, astrocytes, microglia, oligodendrocytes, vascular cells, and many other cell types^1,2^.

Spinal cord injury (SCI) is a traumatic event that can cause long-lasting paralysis, pain, autonomic dysregulation, and body-wide physiological changes^3^. While understanding and developing therapeutics that target cellular changes within the lesion epicenter is undoubtedly valuable, there is an emerging focus on molecular and neural engineering approaches to augment plasticity and reorganization in anatomically incomplete injuries, which are the most common in patients^4–6^. Molecular approaches can induce sprouting of descending spared projections^7,8^ or reorganization of the neural circuits below the injury^4,6^. Neural engineering approaches such as epidural electrical stimulation combined with rehabilitation training can promote impressive gains in motor function and autonomic control and provide enhanced quality of life^9–11^, underscoring the importance of understanding intrinsic potential in the tissue below the injury site. Understanding the cellular mechanisms by which reorganization occurs in spinal cord neurons is a crucial step to promote recovery.

What may be the underlying mechanisms of plasticity in the lumbar spinal cord that enable recovery after injury? There are many forms of plasticity, from synaptic remodeling to local axonal sprouting and long-distance axon growth^12,13^. After injury in the peripheral nervous system, damaged axons can successfully regrow and innervate their targets to restore function^14,15^. However, regeneration in the CNS is generally limited, due to both cell-intrinsic and cell-extrinsic factors^16^. Although most CNS neurons are capable of some plasticity, these processes are limited to local changes such as synaptic remodeling. However, there are a few examples of CNS neurons that can regenerate^17^, suggesting that regenerative capacity is a cell type-specific feature. We hypothesized that the many neuronal subpopulations in the lumbar cord—with their diverse molecular identities^18,19^, physical properties, and connectivity^20^, – may differentially contribute to recovery after spinal cord injury^21^, and that specific neuronal subpopulations may display their own strategies for repair.

Here, we sought to uncover the dynamic cell type-specific responses of the lumbar spinal cord following SCI to identify the cell type-specific molecular and cellular mechanisms that promote or restrict recovery. First, we performed severe thoracic contusion spinal cord injuries in mice and tracked the progression of injury responses from acute to chronic time points. To profile the diverse cell types within the lumbar spinal cord following thoracic injury, we used single nucleus RNA Sequencing (snRNA-seq) and created an atlas of the lumbar cell types after injury (https://seqseek.ninds.nih.gov/spinalcordinjury). This resource reveals both the changes in relative composition of cell types following SCI and the changes in gene expression within each cell type. The size and scope of this dataset allowed identification of rare cellular populations that displayed molecular pathways with the potential to support recovery. Specifically, we identified neuronal populations that expressed regeneration-associated genes (RAGs). These neurons were largely excitatory, and their spatial distribution, as well as gene expression, suggested that they are ascending projection neurons that link the spinal cord and brain. We identified the RAG-expressing neurons to be Shox2-expressing V2d and spinocerebellar neurons. Using viral-labeling strategies, we showed that after thoracic injury spinocerebellar neurons increased axons and collaterals below the injury site, indicating structural remodeling. Together these findings shed light on the limited spontaneous mechanisms of repair in the tissue below the lesion and the latent potential for targeted neuro-regeneration and tissue remodeling therapies.

## Results

### A single-cell atlas of the lumbar cord after spinal cord injury

**Fig. 1:**
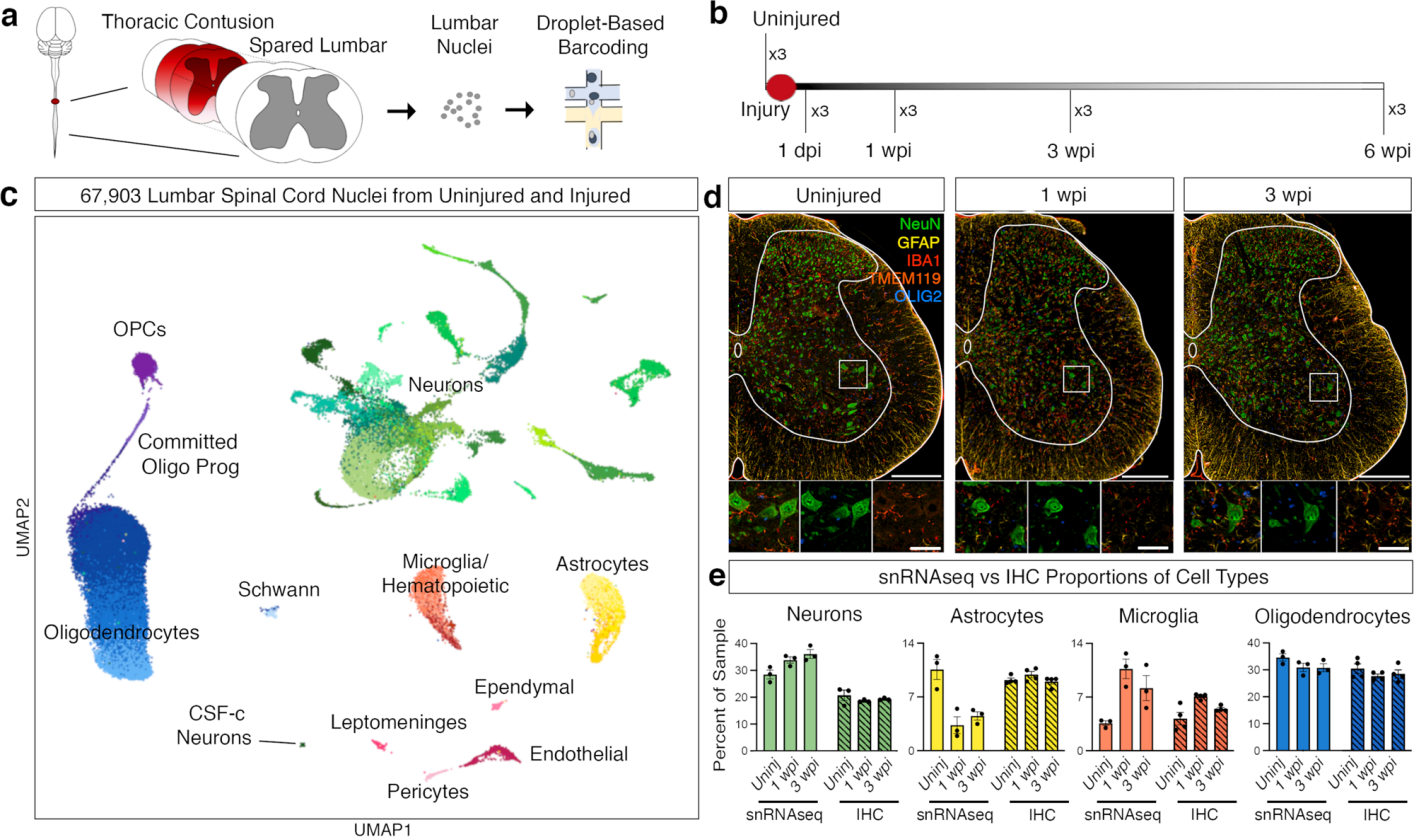
Single nucleus RNA sequencing of the lumbar spinal cord after thoracic contusion. **a** Schematics depicting the experimental design for snRNA-seq, showing the injured thoracic cord and lumbar cord (with dark red representing the lesion) as well as nuclei isolation from the intact lumbar cord followed by droplet-based barcoding for single nucleus RNA sequencing. **b** Top, an overview of experimental design for injury and tissue collection. The lumbar spinal cord of three animals from each time point: uninjured, 1 dpi (day post injury), 1 wpi (week post injury), 3 wpi, and 6 wpi. **c** Uniform manifold approximation and projection (UMAP) visualization of 67,903 nuclei from uninjured and injured lumbar spinal cords, revealing 9 classes and 39 subtypes. Colored by green (neurons), yellow, astrocytes, orange-red (microglia), purple (OPCs), blue (oligodendrocytes), light blue (Schwann), light pink (pericytes), pink (ependymal), magenta (leptomeninges), brick-red (endothelial). **d** Multiplex immunohistochemistry (IHC) of the lumbar spinal cord from uninjured, 1 wpi and 3 wpi. Tissue was stained for NeuN (green), GFAP (yellow), IBA1 (red), TMEM119 (dark orange), and OLIG2 (blue). Scale bars are 200 µm (main) and 50 µm (inset), respectively. **e** Quantification of the proportion of cell types from the snRNA-seq data and immunohistochemistry in tissue. Mean ± SEM; snRNAseq, *N* = 3; immunohistochemistry, *N* = 4. Source data are provided as a Source Data file.

We profiled the cell types in the lumbar cord after spinal cord injury at translationally relevant time points to create an atlas of cell type-specific responses and uncover biological changes. A severe contusion was delivered to the thoracic (vertebral level T9) spinal cord of mice, resulting in paralysis (Supplementary Fig 1, Supplementary Dataset 1). Locomotor function was tracked over a range of time points that, in mice, corresponded to the acute injury period (1 day post injury (dpi)), the subacute and intermediate stages that are typically targeted with therapeutic interventions (1 week and 3 weeks post injury/wpi), and a chronic time point at which recovery typically plateaus (6 wpi; Supplementary Fig 1). We then dissected the lumbar spinal cord of three animals from each time point and performed single nucleus isolation and RNA sequencing (Fig. 1a, b)^22^. We clustered the data, integrating by time point, and removed clusters that were low-quality as well as doublet clusters, yielding a final dataset of 67,903 nuclei (see “Methods” for details).

To create an atlas of cell type responses in the lumbar cord after injury, we first clustered the nuclei at a coarse level to highlight large-scale changes. We identified 9 major cell classes: neurons, astrocytes, microglia/hematopoietic cells, oligodendrocyte lineage cells, Schwann cells, endothelial cells, pericytes, ependymal cells, and leptomeninges (Fig. 1c). Each of these major classes was identified using well-established markers^19^ (Supplementary Fig. 2, Supplementary Dataset 2). Neurons expressed *Snhg11, Rbfox1, Rbfox2, Snap25*. Astrocytes expressed *Slc7a10, Agt, Gfap*, and *Vim*. Microglia/hematopoietic cells expressed *C1qa, Ctss, Gpnmb, Lgals3, Itgax, Ms4a4b, Cd3g*, and *Nkg7*. Oligodendrocyte lineage cells including oligodendrocyte precursor cells (OPCs) expressed *Cspg5* and *Tnr*; committed oligodendrocyte progenitor cells (COPs) expressed *Fyn* and *Tcf7l2*, whereas myelinating and mature oligodendrocytes expressed *Plp1, Mag*, and *Mog*. Schwann cells, which were part of the lumbar spinal cord roots that were in the dissected tissue, expressed *Mpz* and *Pmp22*. Vascular cells included endothelial cells, which express *Bsg* and *Cldn5*, and pericytes, which express *Vtn* and *Pdgfrb*. Ependymal cells expressed *Nnat* and *Dnah12*. Leptomeninges expressed *Dcn* and *Col1a1* (Supplementary Fig. 2, Supplementary Dataset 2). With this approach, we have identified the major cell classes of the uninjured and injured mouse spinal cord.

### Coarse cell types in snRNA-seq and in tissue

We next compared the proportion of the coarse cell types in the snRNA-seq dataset to the proportion of these cell types as detected by immunohistochemistry in tissue sections. In the snRNA-seq data, we calculated the proportion of each cell type within a given sample. In the uninjured tissue, oligodendrocytes represented the largest proportion of the uninjured spinal cord (34.6 ± 1.4%), followed by neurons (29.4 ± 1.7%), astrocytes (10.5 ± 1.3%), and microglia/hematopoietic cells (3.7 ± 0.3%; mean ± SEM, *N* = 3, Fig. 1e). We found that the overall cellular composition stayed relatively stable after injury, with few exceptions. Astrocytes appeared to decrease in proportion at 1 and 3 wpi (*p* = 0.013 and *p* = 0.013) while microglia increased in proportion at 1 wpi (*p* = 0.006) and neurons increased in proportion at 3 wpi (*p* = 0.030).

To determine whether these observations accurately reflect endogenous changes in the lumbar spinal cord, we performed immunohistochemical staining and in situ hybridization experiments in tissue sections from healthy animals, 1 week, and 3 weeks after thoracic contusion injury. We stained for neurons (NeuN), astrocytes (GFAP and SOX9), oligodendrocytes (OLIG2), as well as microglia and macrophages (TMEM119, IBA1, Fig. 1d, e). Quantitative analysis of the multiplexed immunohistochemistry confirmed the snRNA-seq cellular composition. In the uninjured tissue, the proportions of astrocytes, microglia, and oligodendrocytes were not significantly different between the two technical approaches (astrocytes 11.7 ± 0.7%; microglia/hematopoietic 4.7 ± 0.3%; oligodendrocytes 30.5 ± 0.4%; mean ± SEM; *p* > 0.05; Supplementary Dataset 3). However, neurons were represented at a larger fraction in the snRNA-seq dataset compared to in tissue quantification of NeuN-positive cells (neurons 20.8 ± 0.6%, *p* = 0.040). This might reflect a decrease in non-neural cells at 3 wpi or a bias in our snRNA-seq dataset toward neurons, which had higher genes per nucleus.

While astrocytes appeared to decrease at 1 and 3 wpi in the snRNA-seq dataset, this result was not confirmed with immunohistochemistry in tissue (Fig. 1d, e). Rather, we observed no significant change in SOX9-expressing astrocytes over time (Fig. 1e). To further characterize the proportion of astrocytes using combinatorial RNA expression, we performed multiplexed RNA in situ hybridization using *Agt*, *Gja1*, and *Aqp4* markers. In this context, a modest decrease was observed from 11.7% in uninjured lumbar cords to 9.5% 1 wpi (±0.7%, 0.9%, respectively, Supplementary Fig. 3). This suggests that the proportional change in astrocytes that we observed in the single-cell atlas did not reflect endogenous cell type changes, but is likely due to the overall proportional shifts of cell types in the injured spinal cord.

We emphasize the importance of in situ validation for cell proportion changes in single-cell RNA sequencing data to distinguish authentic differences in endogenous cell compositions. While some cell types were significantly different between snRNA-seq and in tissue proportions, the composition of cell types in tissue generally reflected those observed in the sequencing analysis. Overall, single nucleus RNA sequencing provides an unbiased profiling of cell types that reflects in-tissue spinal cord biology.

### Composition and changes within 39 refined cell types

**Fig. 2:**
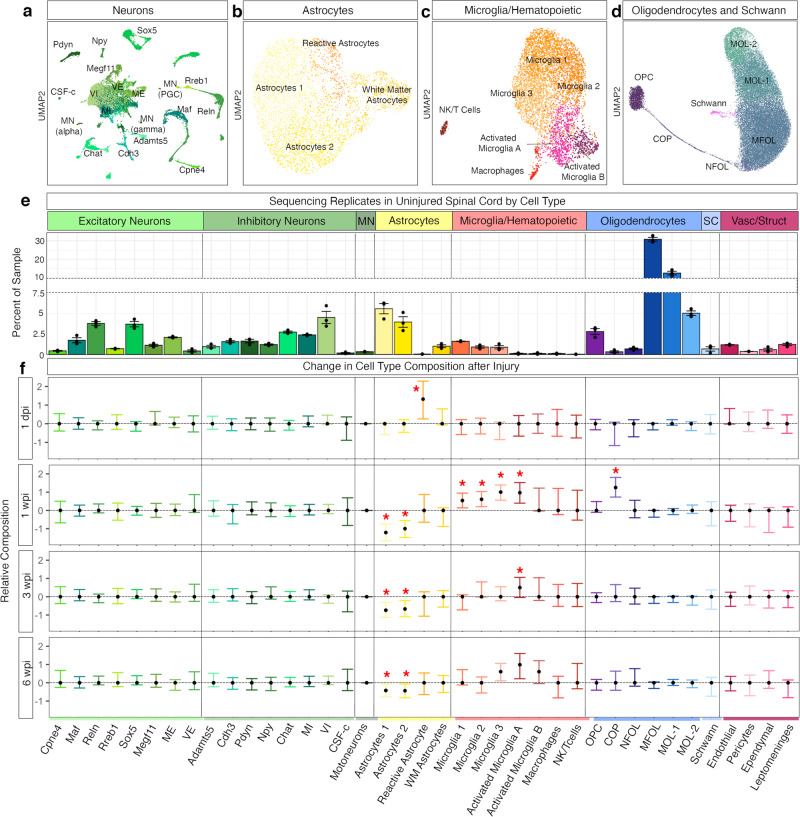
Cell type composition in the uninjured spinal cord and after injury. **a**–**d** UMAPs depicting subclustering of major cell types: **a** neurons, **b** astrocytes, **c** microglia/hematopoietic cells, **d** oligodendrocyte lineage and Schwann cells. **e** A bar plot showing the 39 cell types in the atlas and their relative percent in each sample in the uninjured spinal cord. Individual replicates (*N* = 3) are shown as well as mean ± SEM. **f** The relative composition of the 39 cell types comparing injured samples (1 dpi, 1 wpi, 3 wpi, and 6 wpi) to uninjured, generated using scCODA showing the final parameter output from scCODA (confidence interval shown as 3–97% high-density index around the mean). Cell types with an inclusion probability > 0.85 were deemed significant. Significance depicted with a red asterisk. *N* = 3. For the log FC of individual replicates, see Supplementary Fig. 8. Source data are provided as a Source Data file.

Given the size of this dataset at 67,903 nuclei, we were able to cluster at a higher resolution to identify rare cell types. In this second level of hierarchical clustering, we subclustered neurons, astrocytes, microglia/hematopoietic, and oligodendrocyte lineage/Schwann cells yielding 39 cell types (Fig. 2a–d). Replicates are shown in the uninjured spinal cord by cell type, calculated by the percent within a given sample (Fig. 2e, Supplementary Dataset 3). To assess if the subpopulations increase or decrease after injury, we used scCODA, a Bayesian model for compositional single-cell data analysis (Fig. 2f)^23^. The scCODA framework models cell type counts while considering negative correlative bias via joint modeling of all measured cell type proportions. Here, we highlight findings from the four major cell classes (neurons, astrocytes, hematopoietic, and oligodendrocyte lineage/Schwann cells), including cell markers, composition, and changes after injury.

### Neurons

The 23,651 neurons were subclustered and annotated by a previously established atlas of the lumbar spinal cord neuronal subtypes^19^ using label transfer (Seurat, see “Methods”). This yielded 17 neuronal populations, including 8 excitatory, 8 inhibitory, and motoneurons (Fig. 2a, Supplementary Fig. 2, Supplementary Fig. 4). Excitatory neurons were marked by expression of *Slc17a6*, inhibitory neurons by *Gad1*, *Gad2*, and *Slc32a1*, and motoneurons by *Slc5a7*, *Chat*, and *Prph*. The excitatory neuron families as defined by Russ et al.^19^ are Cpne4, Maf, Reln, Rreb1, Sox5, Megf11, ME (mid-excitatory), and VE (ventral excitatory). Inhibitory neuron families, are Adamts5, Cdh3, Pdyn, Npy, Chat, MI (mid-inhibitory), VI (ventral inhibitory), and CSF-c (cerebral spinal fluid-contacting neurons). The proportions of these neuronal subtypes did not significantly change after injury (Fig. 2f, Supplementary Dataset 3).

### Astrocytes

Astrocyte subtypes were identified by subclustering 4525 nuclei from coarse clustering (Fig. 1c, Supplementary Fig. 3). Astrocytes included five subtypes that putatively reflect two homeostatic populations, white matter astrocytes and reactive astrocytes (Fig. 2b, Supplementary Fig. 3). All astrocytes expressed *Gfap*, *Agt*, and *Gja1*. The two homeostatic populations, “astrocytes 1” and “astrocytes 2” expressed *Gpc5* and *Slc7a10*, while putative white matter astrocytes did not. White matter astrocytes expressed higher levels of *Gfap*, as well as *A2m*, *Cd44*, *C3*, and *Vim*. Reactive astrocytes significantly increase acutely after injury (from 0.3 ± 0.01% of a sample in the uninjured cord to 2.3 ± 1.2% of a sample 1 dpi, *N* = 3, Fig. 2f), and expressed *Lcn2*, *Rgs20*, *Hpgd*, *Serpina3n*, *Iigp1*, *Gbp2*, and *Slc10a6*^24^. Both coarse and refined clustering of astrocytes indicated a decrease in astrocyte proportions (Figs. 1e and 2f); however, these changes were not confirmed with immunohistochemical detection in tissue as discussed above (Fig. 1d, e, Supplementary Fig. 3b). The apparent decrease in astrocytes after injury in the snRNA-seq dataset may be due to overall proportional increases in cell types in the injured spinal cord or selective vulnerability of astrocytes after injury.

### Microglia and hematopoietic cells

To explore microglia and related hematopoietic cell types in greater depth, we independently clustered the 5080 nuclei and observed three homeostatic and two activated microglia populations, a cluster of macrophages, and a cluster of natural killer and T cells (Fig. 2c, Supplementary Fig. 6). Homeostatic microglial clusters were defined by *Cst3*, *C1qa*, *Ctss*, *Hexb*, *Trem2*, *P2ry12*, and *Tmem119* (Supplementary Fig. 2). The activated microglia populations expressed lower levels of *P2ry12* and *Tmem119* and induced expression of the phagocytic markers *Cd68* and *Lyz2*. In addition, “activated microglia A” expressed *Gpnmb*, *Apoe*, *Lgals3*, *Igf1*, and *Spp1*, while “activated microglia B” expressed genes associated with pro-inflammatory microglia, such as *Ccl2*, *Ccl3*, *Ccl4*, and *Lpl*. Macrophages expressed the genes *Mrc1*, *Cd74*, and *H2-Ab1* and did not express the microglia-specific genes *Tmem119* or *P2ry12*. Natural killer and T cells clustered together and expressed the genes *Ms4a4b*, *Cd52*, *Ptprc*, *Nkg7*, and *Cd3g*. All microglia/hematopoietic cell types increased in proportion relative to other cell types at 1 wpi, particularly activated microglia A, which increased 14.8-fold, from 0.1% (±0.04) of all cells in the uninjured cord to 1.6% (±0.60) 1 wpi (Mean ± SEM; Supplementary Fig. 6) as was reflected in the coarse cell type analysis. Both activated microglia subtypes were still present at 6 wpi, suggesting that they may play an ongoing role at chronic time points (Supplementary Fig. 6), even in lumbar tissue distal to the injury.

Notably, the gene expression profile of “activated microglia A” strongly resembled a signature observed recently in postnatal myelin-phagocytosing microglia, in postnatal microglia that can promote SCI repair, and in disease-associated microglia in conditions such as Alzheimer’s disease in the brain and ALS in the spinal cord^25–28^. In addition, recent work examining the lesion site of SCI has identified an “injury associated microglia” cell type with a similar expression profile^29,30^. Pathway analysis of the genes that characterized “activated microglia A” revealed an enrichment of genes associated with (1) phagocytosis (such as *Lyz2*, *Gpnmb*, *Itgax*, and *Cd68*; GO terms: lysosome, *p* = 3 × 10^−13^, antigen presentation, *p* = 0.0003, phagosome, *p* = 0.0363), (2) lipid metabolism (such as *Fabp5*, *Lgals3*, *Apoe*, *Soat1*, and *Abca1*; GO term: lipoprotein, *p* = 0.0041), and (3) secreted proteins (such as *Spp1* (OPN) and *Igf1*; GO term: extracellular secretion, *p* = 0.0282, Supplementary Fig. 6c, Supplementary Dataset 5).

To determine whether the gene expression pattern that we observed corresponded to an in vivo cell type, we performed in situ hybridization in tissue sections of spared lumbar cords, using *C1qa* (a general microglial marker), *Spp1* (OPN) (which marked activated microglia as well as some ventral horn neurons^19,31,32^), and *Gpnmb* (which was specific to activated microglia A). These cells were observed within the white matter of the injured spinal cord, and appeared consistently in small clusters along the putative rubrospinal tract (RST) and the dorsolateral corticospinal tract (CST) in the lateral white matter at 1, 3, and 6 wpi (Supplementary Fig. 6e–h). Interestingly, this region showed loss of longitudinal axons from the descending tracts but also showed no change in the presence of residual myelin (Supplementary Fig. 6i–l). Together, these data suggest that Activated microglia A cells are similar to previously described “DAM/PAM” cells and were found in the white matter of the injured spinal cord, where they may play a role in the phagocytosis of degenerating axons or apoptotic cells^33^.

### Oligodendrocytes and oligodendrocyte lineage cells

Oligodendrocytes and oligodendrocyte lineage cells comprised the largest proportion of the lumbar spinal cord. We subclustered 32,287 oligodendrocyte and oligodendrocyte lineage cells, as well as 315 Schwann cells. Although the Schwann cells in this study are likely from lumbar spinal cord roots that were in the dissected tissue, we included them in the downstream analysis due to the interest in Schwann cells as a source of repair and remyelination after injury^34^. Oligodendrocyte lineage cells, including oligodendrocyte precursor cells (OPCs), expressed *Cspg5* and *Tnr*; committed oligodendrocyte progenitor cells (COPs) expressed *Fyn* and *Tcf7l2*^25,26^ (Fig. 2d). Newly formed oligodendrocytes (NFOL) expressed *Man1a* and *Synpr*, whereas myelinating and mature oligodendrocytes expressed *Plp1, Mag* and *Mog* (Supplementary Figs. 2 and 7a–c). Myelinating oligodendrocytes expressed *Opalin* and *Kirrel3*, representing a transitional population between newly formed and mature oligodendrocytes. Mature oligodendrocytes expressed *Klk6* and *Art3*. Schwann cells expressed *Mpz* and *Pmp22*. The proportion of oligodendrocyte lineage cells did not significantly change after injury with the exception of COPs, which increased fivefold from 0.3% (±0.1) of cells in the uninjured spinal cord to 1.7% (±0.2) of cells at 1 wpi (Fig. 2f, Supplementary Dataset 3). These committed oligodendrocyte precursor cells resemble previously identified “COPs” in the juvenile mouse^25,26^. The expansion of this population after injury indicates the emergence of new myelinating cells^27,28^ or potential roles for COPs themselves^29^.

To determine if the oligodendrocytes identified here were similar to those previously described after spinal cord injury, we compared our oligodendrocyte lineage cells to those identified in Floriddia et al.^30^. In this previous study, the diversity of mature oligodendrocytes was cataloged, including spatial distribution and susceptibility to spinal cord injury. Of interest, this study found MOL2 and MOL5/6 to be enriched in the spinal cord, with MOL5/6 increasing with age and enriched in the spinal cord lesion site. We analyzed how oligodendrocyte subtypes compare in both studies in the different temporal conditions (using Pearson’s correlation of the shared top 2000 variable genes). Oligodendrocytes from both studies correlated by cell type rather than injury (Supplementary Fig. 7). MOL2 from Floriddia et al. correlated most with the mature oligodendrocytes 1 and 2 (MOL-1, MOL2) from this study across injury conditions. MOL2 was enriched in distal areas of the injury site, where Wallerian degeneration took place. Both datasets similarly classified OPCs, COPs, and MFOLs. These similarities support our classification of oligodendrocyte subtypes.

In all, we provide an in-depth analysis of 39 cell types in the lumbar spinal cord and their compositional changes after injury. The relative abundance of most cell types did not significantly change, except for several glial subtypes (Fig. 2f). The increase in reactivity of cells such as reactive astrocytes at 1 dpi, and microglia at 1 and 3 wpi likely reflected a coordinated response by glia to the assault on the lesion site above. By 6 wpi, only the proportion of were astrocytes was significantly different, indicating a relative return to a native state by this chronic time point.

### Cell type-specific changes in gene expression in the lumbar spinal cord after injury

While the overall composition of cell types is relatively stable after injury, we hypothesized that gene expression within cell types would change after injury, endowing particular cell types with new properties and functions. We next analyzed gene expression across cell types to understand how specific cell types change their molecular repertoire after injury. It is important to note that statistical differences in gene expression can largely be driven by the size of the cluster, with larger clusters having the power to resolve more differentially expressed genes. To determine which cell types changed significantly following injury, we implemented Augur, a method to rank responsiveness of cell types in single-cell data that is not biased by cluster size^9^. We applied Augur to the 39 cell types detected in this study, including 17 neuronal clusters, 4 astrocyte clusters, 7 microglial/hematopoietic clusters, 6 oligodendrocyte-related clusters, Schwann cells, endothelial cells, pericytes, ependymal cells, and leptomeninges. A cell type prioritization score was generated from Augur, indicating the responsiveness of cell types after injury. We found that the average cell type prioritization score across all cell types decreased with time after the acute response to injury, suggesting a progressive return to homeostatic conditions across multiple populations (average 0.55 AUC, Supplementary Fig. 9a, Supplementary Dataset 4). At 1 dpi we found that microglia were the most perturbation-responsive cell type (0.86 average AUC for Microglia 1, 2, and 3). These results suggest that microglia could play an important role in the immediate phase after injury, even in spared tissue distant from the site of injury.

To understand the cellular processes driving these changes, we examined differentially expressed genes within these cell types at each stage after injury and performed gene ontology (GO) and pathway analysis (Supplementary Fig. 9, 10, Supplementary Dataset 5). At 1 dpi, microglia/hematopoietic cells changed dramatically and were characterized by a burst of expression of genes related to cell metabolism, which may support the significant expansion in microglial cell numbers that we observed above. Reactive astrocytes also emerged at 1 dpi, expressing pan-reactive genes such as *Lcn2* and *Serpina3n*, as well as markers for pro-inflammatory and neuroprotective astrocytes^24^ such as *Gbp2* and *Slc10a6* (Supplementary Fig. 9b). Similarly, endothelial cells and meninges displayed acute gene expression changes at 1 dpi, particularly in molecules related to structure, cell–cell connections, and extracellular matrix (ECM) adhesion. In addition, nearly all cell types showed increased expression of *Mt1* (metallothionein-1) and *Fth1* (Ferritin Heavy Chain 1) genes which are both involved in binding heavy metals, including iron. This suggests that extravascular blood may be an early environmental cue to reach the tissue of the lumbar cord (Supplementary Fig. 9b).

While non-neuronal populations changed most acutely, neurons displayed more delayed responses. One week post injury, neuronal populations showed altered expression of genes related to cell stress, including oxidation-reduction processes and protein folding stress response, and molecules related to neurotransmission and ion channel activity. Intriguingly, specific populations of excitatory neurons in the dorsal horn and inhibitory neurons within the ventral horn displayed changes in synaptic organization and plasticity-relate genes. This suggests the potential for tailored circuit remodeling. At the same time, oligodendrocytes altered their cellular metabolism molecules and genes related to cell–cell adhesion. Three weeks after injury, multiple neuronal populations continued to show signatures of cell stress and changes in cell–cell contacts, while oligodendrocytes continued to show changes related to cell metabolism and cell–cell adhesion. Finally, by 6 wpi, many cell types showed modest or no changes in gene expression compared to the uninjured state, showing an overall return to baseline expression patterns by this point (Supplementary Fig. 9b).

Together with the cellular composition analysis above, these data comprise a natural history time course of the lumbar spinal cord response to injury. Within each time window or epoch, the community of cell types responds with diverse molecular signatures which can be observed across time.

### Transcriptional changes in neurons of the lumbar spinal cord after injury

**Fig. 3:**
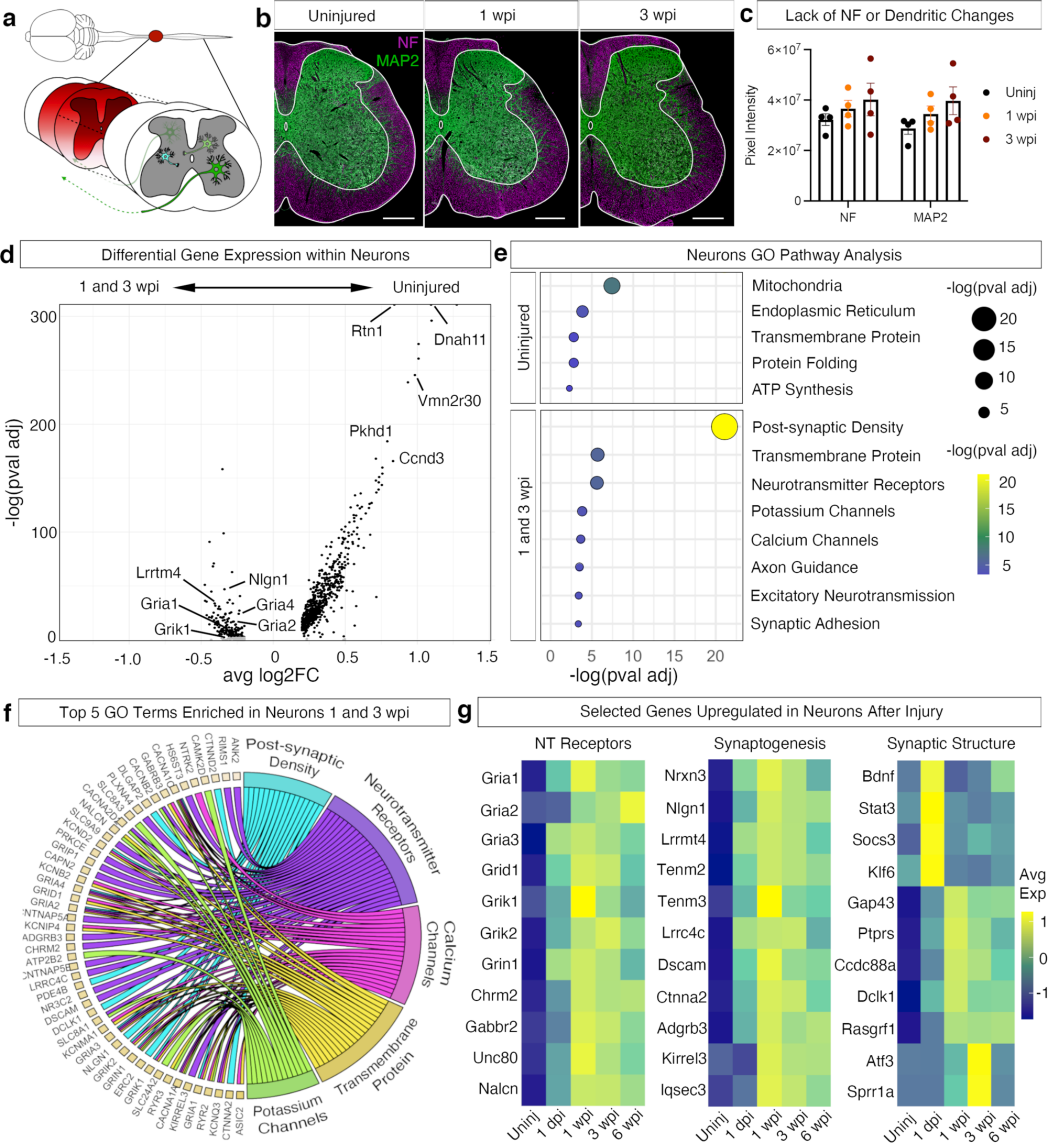
Plasticity-related expression in neurons after injury. **a** Schematic depicting lumbar spinal cord neurons and their response to injury, whether that be an ascending neuron or an interneuron. **b** Immunohistochemistry of the lumbar spinal cord from uninjured, 1 wpi and 3 wpi. Tissue was stained for neurofilament (a cocktail of neurofilament-light, neurofilament-medium, neurofilament-heavy; purple) and MAP2 (green). Scale bars are 200 µm. **c** Quantification of neurofilament and dendritic changes. Pixels were quantified from thresholded images of neurofilament and MAP2. Error bars are mean ± SEM (*N* = 4). **d** Differential gene expression analysis comparing uninjured to 1 and 3 wpi neurons, the time points of maximal neuronal gene expression changes. Black dots indicate *p* value adjusted < 0.001, gray indicate *p* ≥ 0.001. **e** Pathway analysis for differentially expressed genes between uninjured and injured time points. *X*-axis indicates −log(*p* val adj) of GO and KEGG pathway clusters. *P* values (adjusted) were calculated using Benjamini–Hochberg false discovery rate (FDR). Yellow indicates relatively high normalized average expression and dark blue indicates relatively low normalized average expression. **f** Chord plot indicating shared genes between top 5 GO terms from genes upregulated 1 and 3 wpi. **g** Heatmaps showing average neuronal gene expression from top GO terms, including neurotransmitter receptors, synaptogenesis, and synaptic structure. Yellow indicates relatively high normalized average expression (1) and dark blue indicates relatively low normalized average expression (−1).

Neurons in the lumbar cord are largely spared, unlike the neurons at the lesion site^35^. However, many of these cells undergo axotomy and abrupt changes to their descending input^36,37^. We sought to characterize the changes within all neurons after injury. Histological analysis of the tissue, revealed no significant change in the number of neurons (Fig. 1e), neurofilament signal, or MAP2^+^ dendritic signal (Fig. 3b, c) after injury, suggesting no large-scale death or loss of neuronal processes. However, overall, neurons displayed dynamic changes in gene expression, particularly at 1 and 3 wpi, with more genes enriched in uninjured neurons compared to injured neurons (Fig. 3d). Pathway analysis showed that mitochondrial, endoplasmic reticulum, and ATP synthesis pathways are enriched in the uninjured cord (Fig. 3e), likely reflecting a greater level of neuronal firing and homeostatic function. At 1 and 3 wpi, pathways associated with plasticity such as post-synaptic density, neurotransmitter receptors, and axon guidance were upregulated. Particular genes that are upregulated include neurotransmitter receptors (such as *Gria1*, *Gria2, Gria3*, *Grid1, Grik1*, and *Chrm2*), those related to synaptogenesis (*Nrnx3, Nlgn1, Lrrmt4, Tenm2, Lrrc4c*, and *Dscam*), and synaptic structure (*Bdnf, Stat3, Socs3, Klf6, Gap43, Atf* and *Sprr1a*, Fig. 3g). The upregulation of these pathways suggests a broad change in neurotransmission and synaptic remodeling amongst neurons in the first few weeks after spinal cord injury. These changes would likely render spinal neurons more responsive to low levels of neurotransmitters and their activity within local circuits.

### Rare populations of spinal neurons induce a gene expression signature of regeneration

We observed broad changes in the expression of genes related to neural excitability, plasticity, and circuit structure that could support tissue-wide changes in function (Fig. 3). In addition to these broad effects that could alter local spinal network function, we hypothesized that specific neuronal populations might be capable of spontaneous long-range remodeling. Such changes would be challenging to observe without the resolution of single nucleus sequencing, and thus the dataset that we generated held opportunities for discovery.

**Fig. 4:**
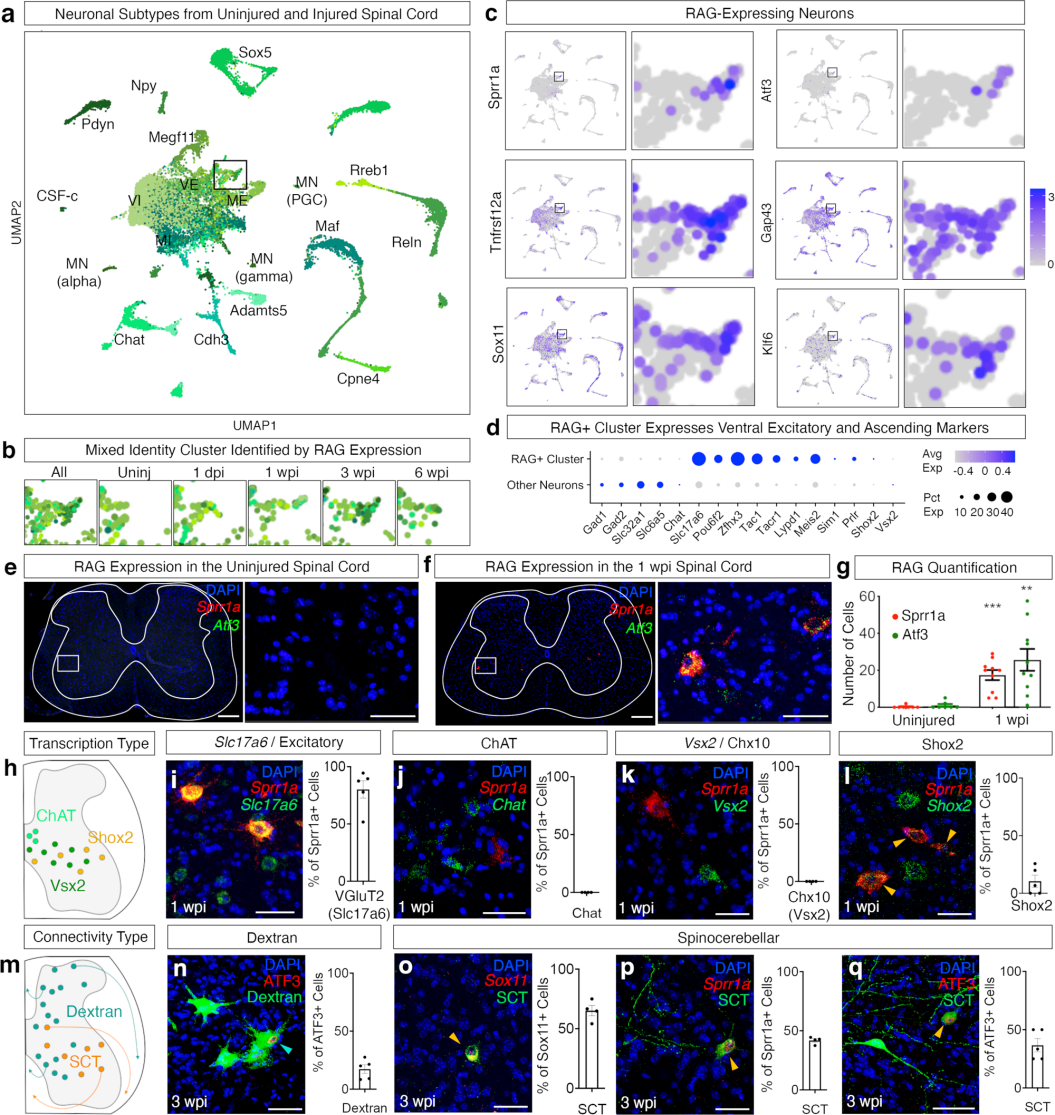
Specific neurons express genes associated with regeneration. **a** UMAP showing predicted neuronal families. **b** Targeted view of RAG-expressing cluster over injury time points. **c** Featureplots showing RAGs expressed in neurons. **d** A dotplot showing expression of RAGs within targeted RAG-expressing cluster (cluster 23 defined by independent clustering in Supplementary Fig. 5). Average expression (avg exp) is indicated by color (gray to blue) and percent expressed (pct exp) is indicated by the size of the circle. **e**, **f** RNAscope in situ hybridization showing expression of *Sprr1a* and *Atf3* in the uninjured cord and 1 wpi. Scale bars are 200 and 50 µm, respectively. **g** Quantification of *Sprr1a*+ cells and *Atf3*+ cells in the uninjured and 1 wpi injury cord (*p* = 0.001 shown as ***p = 0.0037 shown as **, two-sided unpaired t-test, Error bars indicate ± SEM, *N* = 7, 10). **h** Diagram of spatial location of transcription types, including ChAT (light green), Vsx2 (dark green), and Shox2 (orange). **i–l** Visualization and quantification of VGluT2/*Slc17a6*+, *Shox2*+, Chx10/*Vsx*2+, and *Sprr1a*-expressing cells in the ventral spinal cord. Scale bars are 50 µm. Error bars indicate ± SEM (*N* = 5, 4, 4, 6 animals). **m** Diagram of spatial location of connectivity types, including ascending neurons labeled by dextran (aqua) and spinocerebellar (SCT, orange) neurons. **n** Visualization and quantification of lumbar spinal cord neurons labeled by dextran injected into a thoracic contusion lesion site. Aqua arrows indicate ATF3 and dextran overlap. Scale bars are 50 µm. Error bars indicate ± SEM (*N* = 5 animals). Visualization and quantification of the percent of RAG-expressing cells—*Sox11* (**o**), *Sprr1a* (**p**), and ATF3 (**q**) that are spinocerebellar. Spinocerebellar neurons are shown in green and RAGs are shown in red. Orange arrows indicate RAG gene and spinocerebellar dual-labeled cells. Scale bars are 50 µm. Error bars indicate ± SEM (*N* = 4, 4, 5 animals). Source data are provided as a Source Data file.

As was described above, we determined the refined identities of neuronal populations in the dataset using annotations described in a recent atlas of mouse spinal cord cell types^19^. While we observed nearly all the cell types that we expected, one group of neurons remained challenging to categorize (Fig. 4a, b, Supplementary Fig. 5b, c). By clustering neuronal populations without label-transfer annotations, we found that these neurons were distinguished by genes associated with axon regeneration, including *Atf3*, *Sprr1a*, *Tnfrsf12a* (Fn14), *Sox11*, *Klf6, Bdnf*, *Adcyap1*, and elevated expression of *Gap43* (Fig. 4c, Supplementary Fig. 4c, d). These genes all belong to a class of “regeneration-associated genes” (RAGs) that is induced robustly in the peripheral nervous system after nerve injury and can enable regeneration of axons^14,15,38^. The expression of individual regeneration-associated genes has been reported in the spinal cord after injury^38–43^. However, the full spatiotemporal profile of this rare central nervous system phenotype, the cellular identity of these neurons, and the association with axon outgrowth in this context are all unknown. This has been a major obstacle in understanding what enables, restricts, or modulates circuit reorganization after injury.

We examined the expression of cell type marker genes in this RAG^+^ cluster and found evidence of a mixed cell type origin. Cells with the highest RAG expression largely downregulated their endogenous gene expression, as previously reported in peripheral neurons, confounding the initial molecular definition of their cell type^14,15^. Despite this caveat, the RAG^+^ cluster expressed a diverse set of genes associated with ascending projection neurons from the lumbar spinal cord to the brain, including *Lypd1*, *Tacr1*, *Zfhx3*, *Pou6f2*, and *Tac1*^44–47^ (Fig. 4d). Additionally, the expression of *Slc17a6* (vGlut2), *Zfhx3*, and *Pou6f2* suggested that some of these cells were likely excitatory neurons that resided in the lateral part of deep dorsal or ventral horn^19^.

We next probed the expression of *Sprr1a* and *Atf3* in tissue to test whether the RAG signature in the sequencing data is reflected in vivo. Expression of *Sprr1a* and *Atf3* were observed in lumbar spinal cord tissue beginning at 1 week following spinal cord injury and extending to chronic time points at three and 6 weeks after injury (Fig. 4e–g, Supplementary Fig. 5g, Supplementary Fig. 15), thereby confirming the transcriptional data above. Spatial analysis using in situ hybridization revealed a rostral-caudal gradient in the number of RAG-expressing cells, with a greater number of *Sprr1a*-positive cells at rostral lumbar segments closer to the lesion site (Supplementary Fig. 11a, b). The cellular distribution within the transverse plane also shifted such that RAG-expressing cells were found in the dorsal, mid, and ventral horns at L2, but were restricted to the ventral horn in L5. We compared RAG expression to that of the excitatory marker *Slc17a6* and confirmed that most RAG-expressing neurons were indeed excitatory (Fig. 4i), and we next examined whether these represent any of the V0c, V2a, or V2d ventral excitatory populations^48,49^. We did not detect any co-expression of RAGs with the V0c marker *Chat* or the V2a marker *Vsx2*. In contrast, a small subset of RAG neurons expressed *Shox2* (Fig. 4j, k), a marker of V2d excitatory, rhythm-generating central pattern generator neurons of the ventral horn^50–52^.

To determine if certain neurons have a molecular predisposition to express RAGs over others, we applied single nucleus ATAC-seq to the lumbar spinal cord of uninjured mice. We leveraged our snRNA-seq annotations to identify cell types, first assessing neurons compared to non-neurons (Supplementary Fig. 12a–d). We detected increased chromatin accessibility of *Sprr1a, Sox11*, and *Gap43* RAG loci within neurons. To provide greater resolution of neurons, we next grouped these into families of cell types, including dorsal excitatory (DE), dorsal inhibitory (DI), mid-excitatory (ME), mid-inhibitory (MI), ventral excitatory (VE), ventral inhibitory (VI) and motoneurons. We found no clear differential pattern of chromatin accessibility between families of neurons (Supplementary Fig. 12e–h). Although this dataset did not provide deep neuronal subtype resolution, these findings do not support the existence of specific neuronal subpopulations that are primed to express RAG genes.

If molecularly defined populations do not explain the majority of RAG-expressing neurons, what other factors should be considered? Ascending projection neurons are important candidates for RAG expression after injury based on the expression of ascending markers in the RAG^+^ cluster and the passage of these neurons through the lesion area. We next hypothesized that the lumbar projection neurons with axons directly injured by the thoracic contusion respond by expressing this pro-regenerative signature.

To determine whether direct injury to axons elicited RAG expression, we performed complete transection injuries of the thoracic spinal cord plus dextran injection to the transected area (Supplementary Fig. 13a). One week later, we found that 4.6% of neurons in the lumbar cord were labeled by dextran (taken up by cut axons and transported to the cell body), with less than 1% of total neurons expressing ATF3 (Supplementary Fig. 13b, c). Of the ATF3-expressing neurons after complete transection, less than half (48%) were dextran positive (Supplementary Fig. 13b, c). We repeated this study using a lateral hemisection injury to distinguish ipsilateral and contralateral-projecting populations and found similar results (Supplementary Fig. 13d, e). Thus, ATF3 is either expressed by both injured and non-injured neurons or the dextran labeling was not complete in labeling all directly injured neurons. More importantly, we found that only a subset of lumbar projection neurons that were directly injured by transection (and took up dextran label at the injury site) induced expression of ATF3. Finally, we examined what percent of lumbar neurons are axotomized during a contusion, by administering dextran to the contusion site at the time of injury. In a contusion model, we found 3 weeks post injury that 18% of neurons were directly injured ascending neurons, as marked by dextran (18%, Supplementary Fig. 11e). In conclusion, the expression of RAGs after the injury is not found in a single molecularly defined population, nor is it a general feature of axotomized spinal neurons.

### Spinocerebellar neurons express RAGs and display axon sprouting below the lesion

**Fig. 5:**
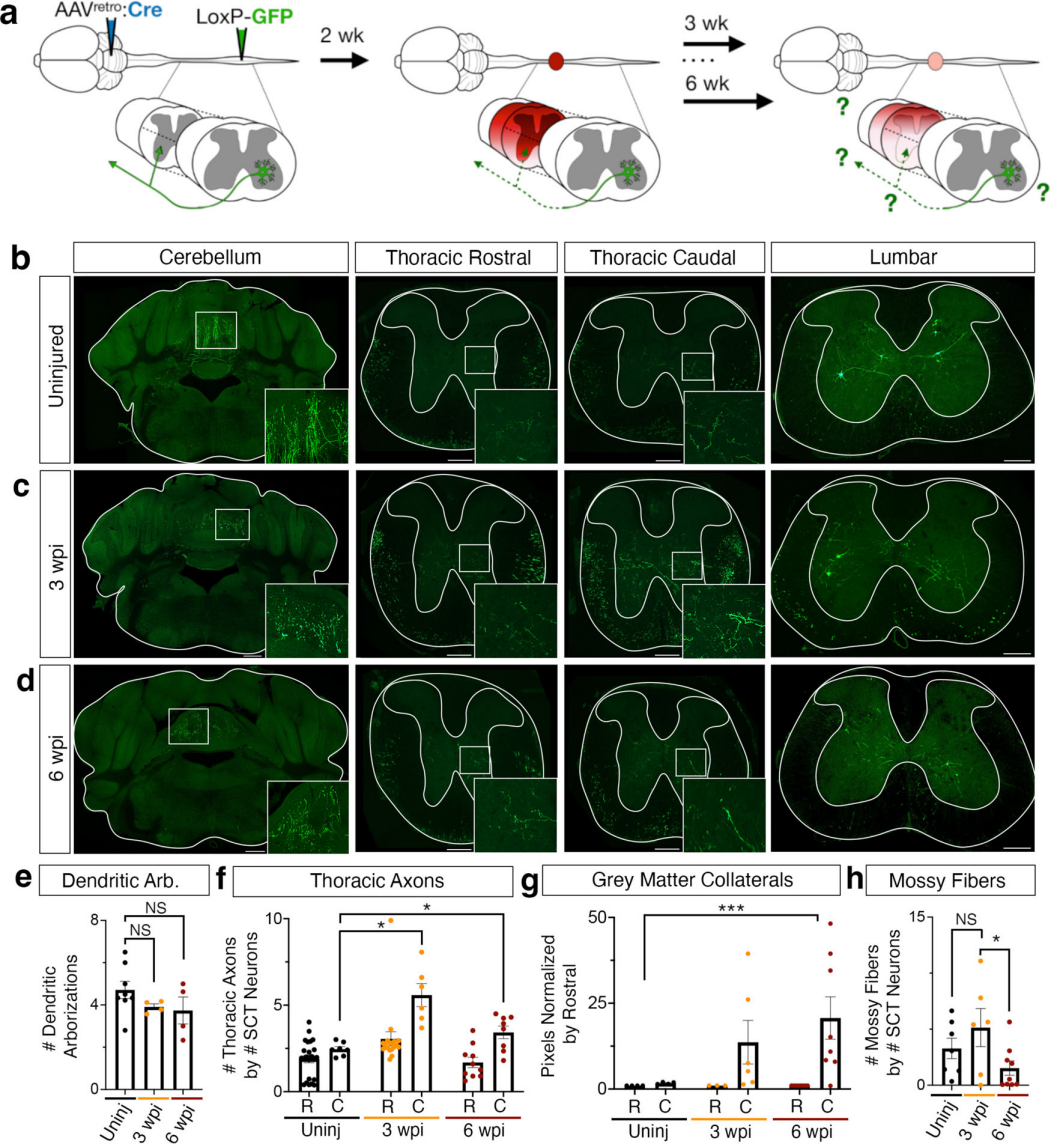
Spinocerebellar neurons express RAGs and display thoracic sprouting after injury. **a** Schematic of AAV- injection and injury. **b–d** Spinocerebellar neurons and their cell bodies, axons, and mossy fibers in the cerebellum, thoracic and lumbar spinal cord. Virus expression is shown in green. Scale bars are 500 µm in the cerebellum and 200 µm for spinal cord sections (thoracic and lumbar). **e** Quantification of dendritic arborizations in SCT neurons (ns, *p* = 0.206, *p* = 0.211, Mann–Whitney test). **f** Quantification of thoracic axons rostral (R) and caudal (C) to the injury site (*p* = 0.0012, *p* = 0.035, Mann–Whitney test). **g** Quantification of gray matter collaterals rostral (R) and caudal (C) to the injury site, as measured by pixels after thresholding (*p* = 0.0485, two-way ANOVA). **h** Quantification of mossy fibers, normalized by the number of SCT neurons in the same animal (*p* = 0.336, *p* = 0.039, two-sided unpaired *t*-test). Mean ± SEM, *N* = 4, 5, 6 animals. *p < 0.05; ****p < 0.0001; n.s. not significant. Source data are provided as a Source Data file.

After the injury, the rare RAG-expressing neurons in the lumbar spinal cord displayed a heterogeneous gene expression pattern. Still, their rostral-caudal distribution, location in the deep dorsal and ventral horn, and excitatory neurotransmitter status suggested that they may be spinocerebellar neurons. Previously, *Shox2* has been reported as a marker of spinocerebellar neurons in the cervical cord^53^. We found that this is not the case in the lumbar spinal cord (0% of spinocerebellar neurons were *Shox2*^*+*^, ±0.0, *N* = 3 animals). To test if these neurons are spinocerebellar, we injected AAV2retro-pmSyn1-EBFP-Cre in the cerebellum and AAV1-Syn-DIO-GFP bilaterally in the lumbar spinal cord to label both ipsi- and contra-laterally projecting spinocerebellar neurons (Fig. 5a). Two weeks later, we delivered a severe thoracic (vertebral level T9) contusion and dextran to label directly injured neurons. After bilateral spinocerebellar labeling and 3 weeks post thoracic injury, we found that 65.2% (±4.3) of *Sox11-*expressing neurons, 41.8% (±1.5) of *Sprr1a*-expressing neurons and 38.0% (±5.6 SEM) of ATF3-expressing neurons were indeed spinocerebellar (Fig. 4o–q, Supplementary Fig 11d, e), while only 1.3% of the spinocerebellar neurons were dextran positive suggesting that spinocerebellar neurons are largely not axotomized by the severe thoracic contusion injury (Supplementary Fig. 11e). Ventral spinocerebellar neurons may avoid direct injury based on the ventral location of their axons. From this data, at least ~55% of ATF3-expressing neurons are ascending, as revealed by dextran labeling (18% directly injured) or spinocerebellar viral labeling (38%). Based on the inefficiencies and variability of these labeling approaches, this likely under-represents the proportion of ascending neurons amongst the RAG^+^ population.

We next asked whether there is there an axon outgrowth or remodeling phenotype that correlates with the RAG gene expression signature that we observed in spinocerebellar neurons? Based on the cell-filling viral label, we examined dendritic arborizations, thoracic axons, collateral axons, and mossy fiber terminals of spinocerebellar neurons after injury and performed this analysis in two independent experimental paradigms: bilateral or contralateral spinocerebellar labeling. Dendritic structure did not change after injury (Fig. 5e, *p* = 0.206, *p* = 0.211, Mann–Whitney test). In contrast, we observed an increase in the number of axons found in the white matter of the thoracic spinal cord below the injury site at both three and 6 weeks after injury (Fig. 5f, *p* < 0.05, *p* < 0.05, Mann–Whitney test) and a dramatic increase in gray matter collaterals of these axons caudal to the injury site (*p* < 0.005, two-way ANOVA). Specifically, these collaterals targeted lamina VII of the ventral horn (Fig. 5b–d). In contrast to these findings, there was no significant change in spinal cord axons in the tissue above the lesion (Fig. 5f, ns). In the cerebellum, there was a trend for an increase in spinocerebellar mossy fibers at 3 weeks after injury (*p* = 0.336, Mann–Whitney test), which then decreased significantly by 6 weeks after injury (Fig. 5g, *p* < 0.05, Mann–Whitney test). This indicates that the terminal synapses of spinocerebellar neurons are largely preserved after spinal cord injury and may display dynamic reorganization. Together, these results show that lumbar spinocerebellar neurons expressed regeneration-associated genes after spinal cord injury, have axons that were spared after a severe contusion, and underwent structural remodeling below the injury site including axonal outgrowth after injury. This highlights an example of spontaneous neuronal remodeling after spinal cord injury, discovered through the power and resolution of single nucleus sequencing.

## Discussion

SCI disrupts neuronal connections, eliciting trauma on a wide array of cells within the tissue. While the capacity for axonal regeneration and recovery after SCI is limited^54,55^, there may be latent mechanisms for spontaneous recovery within the spinal cord, particularly in anatomically incomplete injury where reorganization of circuits below the lesion site can support the restoration of function^54,55^. We used single nucleus RNA sequencing to profile the lumbar spinal cord after a severe thoracic contusion injury to track the cell type-specific injury responses and identify spontaneous changes that could be leveraged for recovery. We present a natural history time course extended from acute through chronic time points and an accompanying interactive website as a resource for the field (https://seqseek.ninds.nih.gov/spinalcordinjury). We observed rare spinal neurons that expressed a pro-regenerative transcriptional signature, identified a major subset of these cells as spinocerebellar neurons and demonstrated axonal sparing and outgrowth of these cells after spinal cord injury.

Our findings build on prior work that used single-cell or nucleus sequencing to profile the cell type-specific responses to spinal cord injury. Most of these studies have focused on non-neuronal cells in and surrounding the lesion area or throughout the spinal tissue, such as the cells that make up the fibrotic core, glial scar, myeloid cells, vascular cells, and oligodendrocyte lineage^30,56,57^. In particular, an emerging finding from multiple studies, bolstered by our own observations, is the presence of damage-associated microglia in disease and injury^56,58–64^. Of the microglia that respond after SCI, activated microglia A cells expressed neuroprotective growth factors and were transcriptionally similar to proliferative axon tract-associated microglia in postnatal mice^33,60,62^, disease-associated microglia^58^, as well as microglia from the human spinal cord^65^. Future work is needed to address the implications of this disease-associated microglia for recovery after injury in adults^61^. While these studies have highlighted the spontaneous trauma responses amongst glia, they have left neuronal plasticity mechanisms largely unaddressed. We previously used single nucleus RNA sequencing to identify neurons that respond to epidural electrical stimulation^66^, but did not examine the effect of spinal cord injury itself or explore the molecular pathways that could mediate spontaneous or therapeutic circuit remodeling.

The cell type- and context-specific factors that control a neuron’s response to injury are not well understood, hampering efforts to target, expand, and modulate this cellular potential. We leveraged the resolution of single nucleus sequencing to explore this question in the context of spinal cord injury. We found that broad neuronal responses mainly included the downregulation of physiological pathways and the upregulation of genes associated with neurotransmission and synaptic structure. In contrast, two distinct subsets of spinal cord neurons—Shox2-expressing V2d neurons and spinocerebellar neurons—expressed RAGs after injury. These genes included transcription factor RAGs (such as *Atf3* and *Sox11*) that act as indirect regulators of many growth proteins, as well as effector RAGs (such as *Sprr1a* and *Gap43*) that play a direct effector role in growth cone and axon outgrowth cytoskeletal changes^40,67,68^. By defining the identities of spinal neurons that express RAGs, our work opens the door to tracking the axonal remodeling that may accompany the transcriptional regeneration signature.

RAGs can be necessary and sufficient to support axon regrowth after injury^14,15,39,40,69,70^, prompting us to test whether spinocerebellar neurons displayed structural plasticity after injury. Indeed, we found that spinocerebellar axons are spared by severe contusion injury and even show increased numbers in the thoracic spinal cord, together with enhanced axon collaterals in the thoracic gray matter and evidence of cerebellar mossy fiber reorganization. Importantly, these structural changes confirm that spinocerebellar neurons, that express RAGs after spinal cord injury, undergo spontaneous axon outgrowth.

What may be the functional consequences of spinocerebellar circuit remodeling? Sprouting of existing axon fibers is an important component of recovery from spinal cord injury, permitting spared neurons to make new connections, serve as circuit relays, and take on compensatory roles for improved behavioral function^13,71–73^. We found that spinocerebellar neurons showed significant remodeling in the caudal thoracic cord just below the lesion, with a major expansion in the lamina VII of the ventral horn. Here, these neurons may contact local central pattern generator circuits or connect with spared and regenerating descending pathways^37,74^. Spinocerebellar neurons also showed structural plasticity at their mossy fiber terminals in the cerebellum. Given the importance of these connections in locomotion and motor learning, such anatomical changes could provide a key substrate for therapeutic interventions^75,76^.

There are several limitations to consider in this work. First, we extracted nuclei instead of whole cells for transcriptional profiling to avoid experimentally induced gene expression and selective cell death common in single-cell profiling^18,32,77,78^. However, this approach may yield fewer genes detected per cell/nucleus and may slightly bias the cellular composition^79^. For example, snRNA-seq on the human brain showed that using nuclei for transcriptional profiling depleted activated microglia, compared to using cells^80^. While it is possible that we under-represented activated microglia in our data, both single-cell and single nucleus RNA sequencing approaches have limitations^81^ that are important to consider when choosing a technique. Second, this study provided a global overview of all cell types in the lumbar spinal cord, and should be followed up by future studies on specific cell types after spinal cord injury enabling deeper analysis of the molecular pathways and trauma responses of each cell type. Third, the atlas component of this work examines changes at the gene expression level and does not address post-transcriptional cellular mechanisms. Despite these limitations, this work provides a powerful and temporally resolved reference atlas of cell type-specific changes after traumatic injury and allowed us to discover rare molecular changes that could provide the substrates of repair and recovery.

Here, we sought to uncover the endogenous mechanisms by which the adult nervous system can recover from a severe SCI. The single-cell atlas, discovery of RAG-expressing neurons, and the plasticity in spinocerebellar neurons following severe but incomplete thoracic contusion injury provide important insight into the natural mechanisms of recovery after SCI. Understanding of these intrinsic mechanisms will provide therapeutic targets to control or even reverse pathological changes across a wide variety of injuries and diseases.

## Methods

### Mice

This study including all procedures and experiments received ethical approval from both the Veterinary Office of the Canton of Geneva (Switzerland) and the National Institute of Neurological Disorders and Stroke Animal Care and Use Committee (ACUC protocol number 1384). Mice for RNA sequencing were female C57BL/6 (12–30 weeks of age). For all other experiments, balanced samples of male and female C57BL/6 mice (12–30 weeks of age) were used. Mice used in this study were housed under a 12-h light–dark cycle (06:00–18:00 light), with ad libitum access to food and water. Room temperature was between 20–26 °C and humidity was between 30–70%.

### Surgical procedures

Surgical procedures were performed as previously described^82^. Briefly, following a mid-thoracic laminectomy (T9 vertebra), a spinal cord impactor (IH-0400 Impactor, Precision Systems and Instrumentation LLC) was used to induce a contusion injury. The applied force was set to 90 kdyn. Spinal transections were performed following a mid-thoracic laminectomy (T9 vertebra), cutting the spinal cord with spring scissors before filling the void with gel foam. Animal care, including manual bladder voiding, was performed twice daily or as needed following injury.

For dextran-labeling experiments, 1 μL dextran (Rhodamine B, 10,000 MW, ThermoFisher Scientific, Catalog Number D1824) was injected into gel foam separating the transected cord.

Two days after injury, all mice were evaluated in an open field, and all animals exhibiting any hindlimb movements were not further studied. For single nucleus RNA sequencing experiments, a larger cohort of mice was taken through kinematic analysis, and three mice representative of each time point were selected. For histology experiments, at least four mice were used for each condition. Mice with bone-hit contusions or injuries that fell a standard deviation outside of the average behavior for each time point were excluded. Due to animal care requirements injury experiments were not performed blinded.

### Viruses

AAV2retro-pmSyn1-EBFP-Cre virus was produced at Addgene (Plasmid #51507)^83^. AAV1-Syn-DIO-GFP virus was produced by Vigene (CV17077-AV1). Viral particles were injected at a titer of 5^E12^−1^E13^ genome copies per mL.

### Intraspinal injections

Intraspinal injections were performed as previously described^84,85^. Briefly, mice were anesthetized by intraperitoneal injection of a drug cocktail containing fentanyl (0.2 mg kg), dexmedetomidine (1 mg/kg), and midazolam (5 mg/kg) dissolved in saline. For spinal injections, a small incision was made in the skin, and the underlying musculature and adipose tissue were teased apart to reveal the vertebral column. Tissue joining the dorsal processes of consecutive vertebrae was removed, and the vertebral surfaces were cleaned with fine forceps and gently separated to reveal the dorsal surface of the spinal cord (at spinal levels L2 and L5). The dura was punctured by pinching with sharp forceps to facilitate the smooth entry of the needle. A glass pulled needle was lowered to a depth of 300 μm, halfway between the midline of the spinal cord and the lateral edge. The needle was then pulled back to 250 μm before pressure injecting 250 nL of viral particles at a rate of 75 nL per minute. Following virus injection, the needle was left in place for one minute before it was removed. Each spinal cord received three unilateral injections at L2, L3/4, and L5^86^.

The overlying muscle was sutured after injections, and the skin incision was closed using wound clips. Anesthesia was reversed by intraperitoneal administration of buprenorphine (0.1 mg/kg), atipamezole (2.5 mg/kg), and flumenazil (0.2 mg/kg) in saline. Additionally, mice received an intradermal injection of meloxicam SR for analgesia and were returned to their home cages.

Mice were excluded when viral labeling showed less than 20 cells in the lumbar spinal cord.

### Intracranial injections

Mice were anesthetized by intraperitoneal injection of a drug cocktail containing fentanyl (0.2 mg kg), dexmedetomidine (1 mg/kg), and midazolam (5 mg/kg) dissolved in saline. A small incision was made in the scalp, and a craniotomy was made at −5.8 mm AP, −4.0 mm ML, referencing from bregma (1Cb-4/5Cb)^87^. Virus (500 nL volume) was pressure injected through a pulled glass needle at a rate of 150 nL per minute, starting at a depth of 1.8 mm, slowly raising to a depth of 1.5 mm. Following virus injection, the needle was left in place for one minute before it was removed. The craniotomy was closed with gel foam followed by bone wax, and the scalp was closed with a wound clip. Anesthesia was reversed by intraperitoneal administration of buprenorphine (0.1 mg/kg), atipamezole (2.5 mg/kg), and flumenazil (0.2 mg/kg) in saline. Additionally, mice received an intradermal injection of meloxicam SR for analgesia and were returned to their home cages.

### Behavioral assessments

All procedures used for mice in the sequencing experiment have been described in detail previously^82^. Limb movements were evaluated while running on a horizontal walkway. Bilateral leg kinematics were captured with the Vicon Motion Systems, UK (combining 12 infrared cameras) for tracking with reflective markers on the crest, hip, knee, ankle joints, and distal toes. The limbs were modeled as an interconnected chain of segments. Based on these, a total of 80 gait parameters were computed for each limb for each gait cycle. All gait parameters are reported in Supplementary Dataset 1. In Supplementary Fig. 1, quantifications of key gait parameters are shown: Step height (calculated from the toe), % drag (as percent of the gait cycle), whole limb oscillation (difference between maximum and minimum angle of limb axis, that is crest to toes, in XY plane within a gait cycle), whole limb oscillation velocity (velocity of the previous), ankle/knee/hip joint oscillation (difference between maximum and minimum joint angle within a gait cycle) and ankle joint oscillation velocity (velocity of previous). Differences among groups were calculated using two-tailed *t*-tests (unpaired) and were considered significant if *p* < 0.05. Data are represented as mean ± SEM unless otherwise indicated. Statistical analyses were performed using GraphPad prism software.

### Analysis of kinematic data

A total of 78 gait parameters were computed for each limb for each gait cycle. We chose to represent the following gait parameters: step height, drag percentage, amp limb, amp speed limb, amp join 1, amp joint 2, amp joint 3, and amp speed joint 3. Differences among groups were calculated using two-tailed *t*-tests (unpaired) and were considered significant if *p* < 0.05. Data are represented as mean ± SEM unless otherwise indicated. Statistical analyses were performed using GraphPad Prism software.

### Nuclei isolation

Nuclei were isolated from adult mouse lumbar cords with proximal dorsal and ventral roots attached using a triton-based protocol adapted from Matson et al.^22^. Briefly, mice were euthanized according to IACUC guidelines. The spinal cord was rapidly dissected and frozen on dry ice. Later, fresh frozen lumbar cords (spinal segment L2-S1) were placed in a Dounce homogenizer (Kontes Dounce Tissue Grinder) containing 500 μL of lysis buffer (0.32 M sucrose, 10 mM HEPES [pH 8.0], 5 mM CaCl_2_, 3 mM MgAc, 0.1 mM ETDA, 1 mM DTT, 0.1% Triton X-100). The cords were dounced with 5 strokes of pestle A, then 5-10 strokes of pestle B. The lysate was diluted in 3 mL of sucrose buffer (0.32 M sucrose, 10 mM HEPES [pH 8.0], 5 mM CaCl_2_, 3 mM MgAc, 0.1 mM ETDA, 1 mM DTT) and passed over a 40 μm strainer. The filtered lysate was centrifuged at 3200 × *g* for 10 min at 4 °C. After centrifugation, the pellet was resuspended in sucrose buffer and incubated for 2 min on ice. The sample was transferred to an Oak Ridge tube and homogenized for 1 min using an Ultra-Turrax Homogenizer (IKA). Then, 12.5 mL of density sucrose buffer (1 M sucrose, 10 mM HEPES [pH 8.0], 3 mM MgAc, 1 mM DTT) was layered below the sample. The tube was centrifuged at 3200 × *g* for 20 min and the supernatant immediately poured off. The nuclei on the side of the tube were resuspended with 100 μL of PBS with 0.04% BSA and 0.2 U/μL RNase inhibitor. Nuclei were inspected for visual appearance and cell lysis using trypan blue and quantified with a hemocytometer before being adjusted to a concentration of 1000 nuclei/μL.

### Single nucleus RNA sequencing

Single nucleus RNA sequencing was carried out using single-cell gene expression 3′ v2 kit on the Chromium platform (10X Genomics) according to the manufacturer’s instructions with one modification. Following reverse-transcription, an additional PCR cycle was added to the number of cycles for cDNA amplification to compensate for decreased cDNA abundance in nuclei compared to cells. Approximately 8000–9000 nuclei were loaded in each well and 3000–7000 nuclei were recovered per sample.

Libraries were sequenced to a minimum depth of 20,000 reads per nucleus using an Illumina HiSeq 3000 (PE 26–8–98 bp). Raw sequencing reads were demultiplexed, aligned, and a count matrix was generated using CellRanger. For alignment, introns and exons were included in the reference genome (mm10).

### Clustering

Analysis was performed in R (version 3.6.1), using the following packages: Seurat (version 3.2.2), RColorBrewer (version 1.1-2), ggplot2 (version 3.3.2), ggrepel (version 0.8.2), dbplyr (version 1.4.4), hrhrthemes (version 0.8.0), plyr (version 1.8.6), viridis (version 0.5.1), tibble (version 3.0.3) and tidyverse (version 1.1.0).

Seurat v3.2.2 was used to filter, normalize, anchor, and cluster the dataset^88^. We filtered nuclei for downstream analysis and included only those with greater than 200 genes detected per nucleus and less than 20% of reads coming from mitochondrial genes. For neurons, the minimum threshold was increased to 500 genes per nucleus as we have previously found that more genes are detected per nucleus in neurons compared with other cell types^18,19^. All genes analyzed were present in greater than three nuclei.

We performed integration of the five conditions (three samples each) using the uninjured samples as a reference, followed by standard log normalization and scaled the data using 2000 most highly variable genes, while regressing out percent mitochondria and nUMI. We used principal component analysis for dimensionality reduction. The number of principal components was selected based on elbow plot inflection, jackstraw plot significance, and PC heatmaps (inspecting gene loadings in each PC and their patterns) for individual principal components. Clustering was performed at two levels—first we performed coarse clustering using 25 PCs and a resolution of 3. After coarse clustering, remaining nuclei were re-normalized, scaled, and 50 principal components were used for dimensionality reduction, with a resolution of 3.

Clusters were visualized using Uniform Manifold Approximation and Projection for Dimension Reduction (UMAP), and cluster markers were found using the “auroc” test in Seurat. Clusters with less than 3 significant markers and had low nUMI, or that were not defined by a cohesive set of genes and had low nUMI, were identified as low-quality clusters and discarded from downstream analysis (Supplementary Fig. 14). We used clustering to identify doublets, rather than defining them on a cell-by-cell basis to avoid discarding cells that may have hybrid or continuum status between two related cell types. All clusters were tested for potential doublet status by examining marker lists for defining genes, using DoubletFinder^17^, and by looking for co-expression of the top markers using FeatureScatter (using top ten cell type markers from a previously published spinal cord atlas^19^). If these analyses supported a doublet identity for the cluster, it was removed from downstream analysis. Overall, 22,435 nuclei were discarded as low-quality or doublets. We next subclustered the neurons, oligodendrocytes, astrocytes, and microglia independently. Raw data for the nuclei in each class was re-analyzed with standard log normalization and a new principal component analysis. We used the following principal components and resolution for each subclustering: neurons: 40 dimensions and resolution 1; oligodendrocytes: 16 dimensions and resolution 0.3; astrocytes: 8 dimensions and resolution 0.3; and microglia: 11 dimensions and resolution 0.8. In addition, we also used label transfer^88^ to analyze the neurons and oligodendrocytes, as described in the Supplemental data. Nuclei had on average 1,392 genes per nucleus in neurons and 471 genes per nucleus in non-neuronal cells (Supplementary Dataset 7).

### Single nucleus ATAC sequencing

Single nucleus ATAC sequencing was carried out using Chromium Next GEM Single Cell ATAC v.1.1 kit on the Chromium platform (10X Genomics) according to manufacturer’s instructions. Libraries were sequenced to a minimum depth of 10,000 reads per nucleus using an Illumina MiSeq (PE 50–8–16–50 bp). Raw sequencing reads were demultiplexed, aligned, and a count matrix was generated using CellRanger-atac 2.0. Cell type-specific dimensionality reduction and cluster analysis for snATAC-seq was performed using ArchR (version 1.0.1). To cluster our scATAC-seq data (for both broad clustering and neuronal subclustering), we used ArchR’s addIterativeLSI function to perform iterative LSI clustering.

Cell types were determined using Seurat’s label-transfer algorithm with cell type annotations in snRNA-seq cells as the reference. Neurons were further subclustered and annotated into “families” (DE, DI, ME, MI, VE, VI, MN) using Seurat’s label-transfer algorithm.

Gene activity scores were calculated using ArchR v1.0.1 with default parameters by using a distance-weighted accessibility model that aggregates signal inside the gene body and in the local genomic region^89^. The resulting gene activity scores were additionally imputed using MAGIC^90^ to reduce sparsity noise in the scATAC-seq data. For peak calling and sequencing tracks, we used the addReproduciblePeakSet function from ArchR (v.1.0.1) with default parameters to call accessible chromatin peaks using MACS2 (v.2.2.7.1) in each cell type subcluster. Marker peaks were identified using ArchR’s getMarkerFeatures function. Sequencing tracks were created using ArchR’s plotBrowserTrack function. All tracks show data that have been normalized by ‘reads-in-TSS’ to account for differences in signal-to-background ratios across samples, unless otherwise stated. For all tracks, genes on the plus strand are shown in red and genes on the minus strand are shown in blue.

### Cell type prioritization by AUGUR and GO analysis

Augur was implemented as previously described using default parameters to rank which cell types changed the most after injury^66^. This approach uses a random forest classifier on subsampled matrices and reports the mean cross-validation AUC across many small subsamples (code is available on GitHub, see below). (The AUC is a measure of the performance of a classifier, with 1 being a perfect classification, 0 being random and negative values indicating poor performance.) For pathway analysis, differential gene expression across conditions was generated using FindMarkers using the Wilcox test. GO Analysis was done using all differentially expressed genes with p_adj < 0.05 using medium stringency and default parameters at https://david.ncifcrf.gov/. GO biological process, cell compartment, and molecular function were analyzed and the clustering annotation tool was selected. Only clusters with an enrichment score (−log of *p* value) greater than 1.3 were considered. In cases in which multiple clusters had the same genes and similar terms, only the most significant is shown.

### Immunohistochemistry and in situ hybridization

Animals were euthanized with avertin and perfused with PBS and then 4% paraformaldehyde. Spinal cords were dissected, fixed in 4% paraformaldehyde overnight, washed in PBS for one hour, then dehydrated in 30% sucrose an additional night before being embedded in OCT.

Immunohistochemistry was performed as previously described^18^. Briefly, spinal cords were cut at 50 µm, placed in blocking buffer (1% IgG-free BSA, 10% normal donkey serum, 0.1% Triton X-100 in PBS) for one hour prior to incubation in blocking buffer and primary antibody for 48 h at 4 °C. Primary antibody was washed off three times in PBS before a 2-h incubation in secondary antibody at room temperature. The secondary antibody was washed off three times in PBS before adding a coverslip.

Multiplex immunohistochemistry was performed as previously described on 10-µm-thick tissue sections^91^. In situ hybridization was performed according to the manufacturer’s instructions for fixed frozen tissue RNAscope (ACD Bio).

### Immunohistochemistry antibodies

#### Primary antibodies

IBA1 (dilution 1:100, Cedarlane Labs, 234006(SY)), TMEM119 (dilution 1:100, Cedarlane Labs, 400004(SY)), CD11c (dilution 1:100, GeneTex, GTX74935), Myelin-MBP (dilution 1:100, BioLegend, 808402), NF-L (dilution 1:200, Cell Signaling, 2835S), NF-M (dilution 1:200, Cell Signaling, 2838 S), NF-H (dilution 1:200, Cell Signaling, 2836 S), NeuN (dilution 1:500, Millipore Sigma, ABN90P), CD68 (dilution 1:100, Abcam, ab125212), CNPase (dilution 1:200, Millipore Sigma, MAB326), GFAP (dilution 1:500, Agilent/Dako, Z033429-2), Cleaved Caspase 3 (dilution 1:100, Cell Signalling Tech, 9661L), and Phospho-IGF1R (dilution 1:100, Invitrogen, PA5-104773).

#### Secondary antibodies

DAPI (dilution 1:1000, Thermo Fisher Scientific, 62248), Goat anti-Hamster IgG Alexa Fluor 647 (dilution 1:200, Jackson ImmunoResearch, 127-605-160), Goat anti-Rabbit IgG Alexa Fluor 430 (dilution 1:200, Thermo Fisher Scientific, A11064), Goat anti-Rabbit IgG Alexa Fluor 430 (dilution 1:200, Thermo Fisher Scientific, A11064), Goat anti-Rabbit IgG Alexa Fluor 594 (dilution 1:200, Thermo Fisher Scientific, A11037), Goat anti-Mouse IgG1 Alexa Fluor 488 (dilution 1:200, Thermo Fisher Scientific, A21121), Goat anti-Mouse IgG1 Alexa Fluor 647 (dilution 1:200, Thermo Fisher Scientific, A21240), Goat anti-Mouse IgG2a Alexa Fluor 546 (dilution 1:200, Thermo Fisher Scientific, A21133), Goat anti-Mouse IgG2b Alexa Fluor 647 (dilution 1:200, Thermo Fisher Scientific, A21242), Goat anti-Guinea Pig IgG Alexa Fluor 546 (dilution 1:200, Thermo Fisher Scientific, A11074), Goat anti-Guinea Pig IgG Alexa Fluor 594 (dilution 1:200, Thermo Fisher Scientific, A11076), Donkey anti-Chicken IgG IRDye 680LT (dilution 1:200, Li-Cor Biosciences, 926-68028), Donkey anti-Rabbit IgG IRDye 800CW (dilution 1:200, Li-Cor Biosciences, 926-32213), Donkey anti-Chicken IgG Alexa Fluor 488 (dilution 1:500, Jackson ImmuoResearch, 703-545-155), and Donkey anti-Guinea Pig IgG Alexa Fluor 647 (dilution 1:500, Jackson ImmuoResearch, 706-605-148).

### RNAscope in situ hybridization probes

ACDbio RNAscope probes: Spp1 (435191), Vsx2 (438341), Gap43 (318621), Chat (408731), Itgax (311501), Mdga1 (546411), Sprr1a (426871-C2), Vgf (517421-C2), Igf1 (443901-C2), Sprr1a (426871-C2), C1qa (441221-C2), Megf11 (504281-C2), GFP (409011-C2), Apoe (313271-C3), Tnfrsf12a (429311-C3), Atf3 (426891-C3), Gpr83 (317431-C3), Pdgfra (480661-C3), Shox2 (554291-C3), Gpnmb (489511-C3).

### Imaging

Images of immunohistochemistry and in situ hybridization samples were imaged using a Zeiss 800 LSM confocal microscope. For quantification, a tile scan image spanning the section was generated for ≥ 3 sections from ≥ 3 mice. Brightness and contrast were adjusted in Photoshop (Adobe), standardized across images.

### Quantification of cell counts from images of immunohistochemistry

Quantification of NeuN, Olig2, and DAPI immunohistochemistry for Fig. 1 was done through a custom MATLAB-based image analysis program (code is available on Github, see below). The software automatically identifies and counts cells based on a criterion that constrains size at a user-selectable intensity threshold. A manual selection tool is also available to identify additional cells that are more difficult to detect. A second channel of the same image shows can be used to count cells that are labeled with both stains using the results from the first channel and a second set of user-selectable thresholds.

Quantification of all other immunohistochemistry was done using FIJI (ImageJ) and photoshop counting tools.

### Pixel quantification

Pixels were quantified using a custom python script (code is available on Github, see below), after a standardized thresholding of images using FIJI (ImageJ).

### Fluorescence intensity

Mean fluorescence intensity was quantified using FIJI (ImageJ) after manually drawing borders based on DAPI and NeuN expression.

### Histological quantification

Two-tailed Mann–Whitney *t*-tests (unpaired) were used for quantification of immunohistochemistry and in situ hybridization. Differences among groups were considered significant if *p* < 0.05. Data are represented as mean ± SEM unless otherwise indicated. Statistical analyses were performed using GraphPad Prism software (version 9.0.0). In all plots, dots represent individual mice. Imaging and most measurements were not done blinded. Only the quantification of pixels for gray matter collaterals was blinded.

### Statistics and reproducibility

Sample sizes are estimated based on previous studies, with at least four animals per condition for anatomical and histological work, and three animals per condition for snRNAseq. For anatomical and histological work, four animals per condition enabled statistical analysis despite a small amount of variability expected after a spinal cord contusion injury. For snRNAseq, three animals were chosen as the minimum number to allow statistical analysis of each time point, while balancing sequencing costs.

AAV injections were replicated in two completely independent experimental cohorts. Mice were randomly assigned to injury groups and AAV-injection groups in each cohort, with AAV injections occurring 2 weeks prior to the injury surgery. Both cohorts were included in the final analysis.

### Reporting summary

Further information on research design is available in the Nature Research Reporting Summary linked to this article.

## Supplementary information

Supplementary Information

Description of Additional Supplementary Files

Supplementary Dataset 1

Supplementary Dataset 2

Supplementary Dataset 3

Supplementary Dataset 4

Supplementary Dataset 5

Supplementary Dataset 6

Supplementary Dataset 7

Reporting Summary

## Data Availability

A searchable version of this data is available at https://seqseek.ninds.nih.gov/spinalcordinjury. Raw sequencing data and count matrices have been deposited to the Gene Expression Omnibus (GSE172167). Source data are provided with this paper.

## Source data

Source Data

## Code availability

Custom MATLAB-based code for quantification of cell counts is available at https://github.com/ArielLevineLabNINDS/CellCounter (10.5281/zenodo.6967482). Custom python-based code for quantification of pixels on thresholded images is available at https://github.com/ijhua/pixel_counts. Code for Augur is available at https://github.com/neurorestore/Augur.
