# Supplementary material for: Single cell atlas of spinal cord injury in mice reveals a pro-regenerative signature in spinocerebellar neurons
Source: Nat Commun. 2022 Sep 26;13:5628. doi: 10.1038/s41467-022-33184-1 (PMC9513082; doi:10.1038/s41467-022-33184-1)
Supplement: Supplementary file 1 — Supplementary Information [file 41467_2022_33184_MOESM1_ESM.pdf]

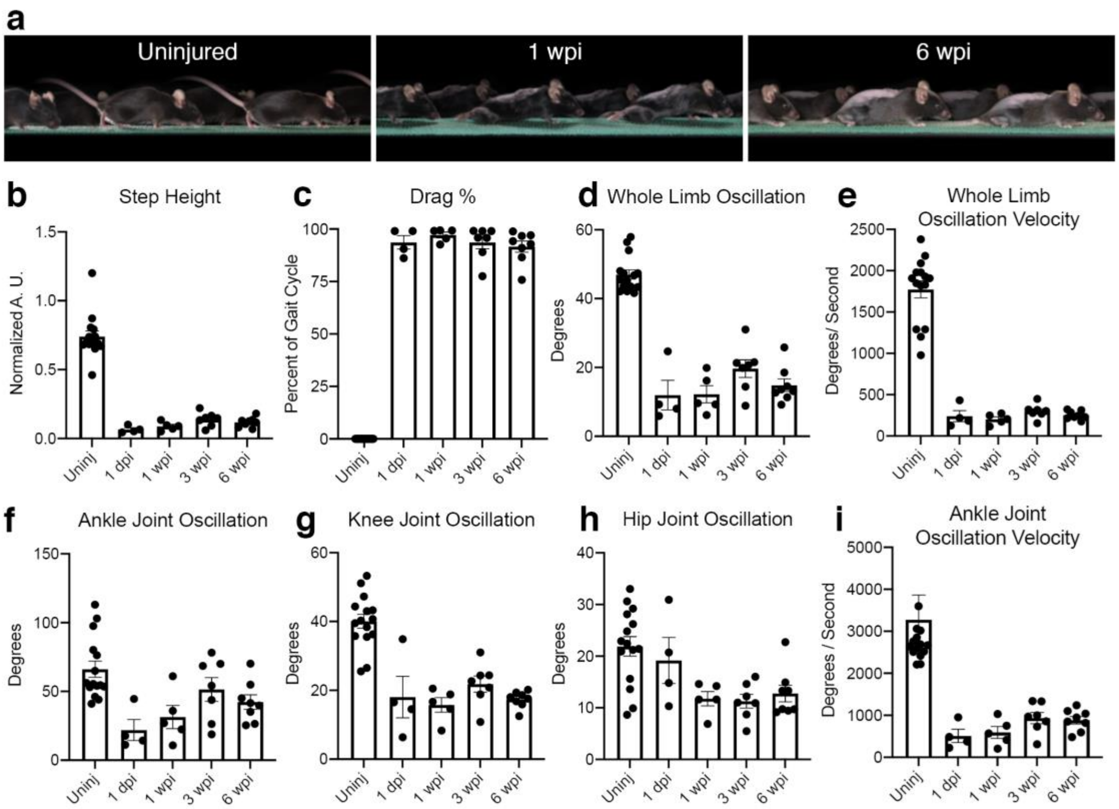

Supplementary Fig. 1. Kinematics of Mice after Injury. a. Chronophotography showing the overground walking of uninjured mice and those 1 and 6 wpi while running on a horizontal walkway. **b-i.** Bilateral leg kinematics, showing **b.** step height, **c.** percent of steps with drag, **d.** whole limb oscillation, **e.** whole limb oscillation velocity, **f.** ankle joint oscillation, **g.** knee joint oscillation, **h.** hip joint oscillation, and **i.** ankle joint oscillation velocity. Error bars indicate ± SEM; Uninj N = 15; 1 dpi N = 4; 1 wpi N = 5; 3 wpi N = 7; 6 wpi N = 8 animals. Source data are provided as a Source Data file.

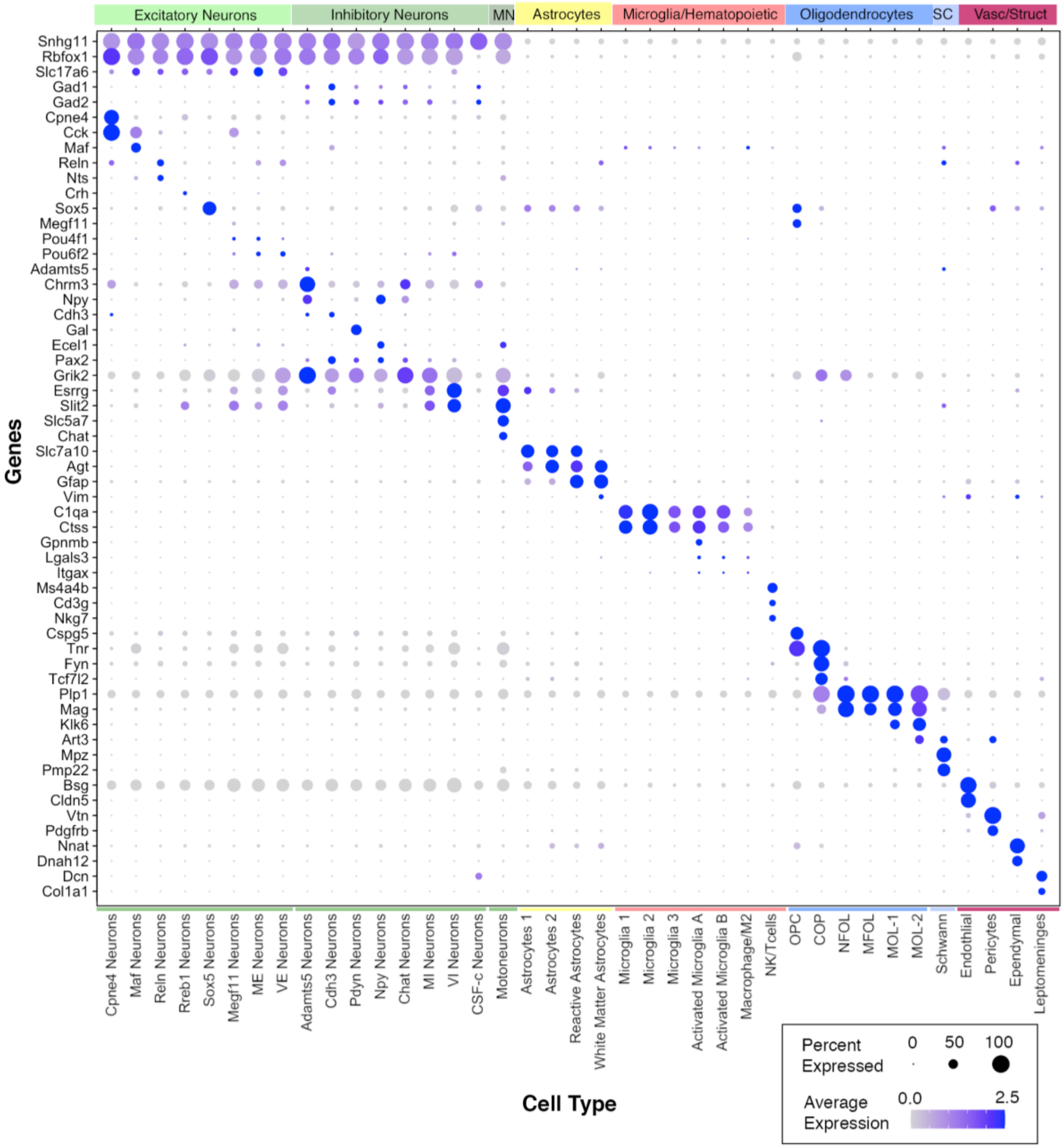

Supplementary Fig. 2. Top Markers for Clusters. Dotplot showing top markers for 39 clusters, from all uninjured and injured timepoints combined. Dot size indicates percent expressed and dot color indicates average expression.

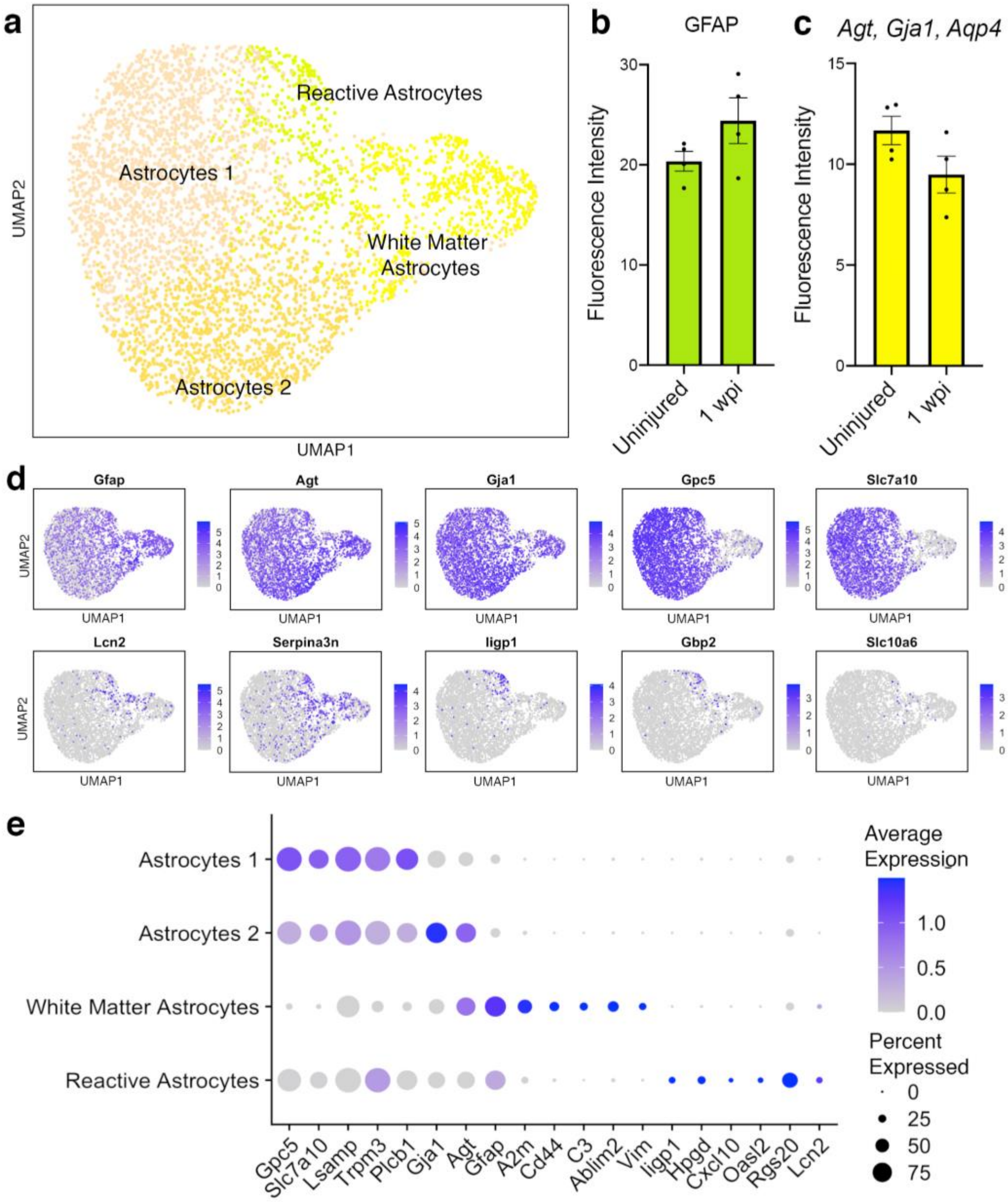

Supplementary Fig. 3. Astrocyte Subclusters. a. UMAP showing astrocyte subtypes, including astrocytes 1, astrocytes 2, white matter astrocytes and reactive astrocytes**. b-c.** In-tissue quantification of markers for astrocytes, including **b.** immunostaining for GFAP and **c.** RNAscope *in situ* hybridization for *Agt, Gja1 and Aqp4*. No significant difference between conditions. Mean ± SEM; N = 4 animals. Source data are provided as a Source Data file. **d.** Featureplots of markers for astrocyte subtypes. **e.** Dotplot showing top markers for astrocytes 1, astrocytes 2, white matter astrocytes and reactive astrocytes.

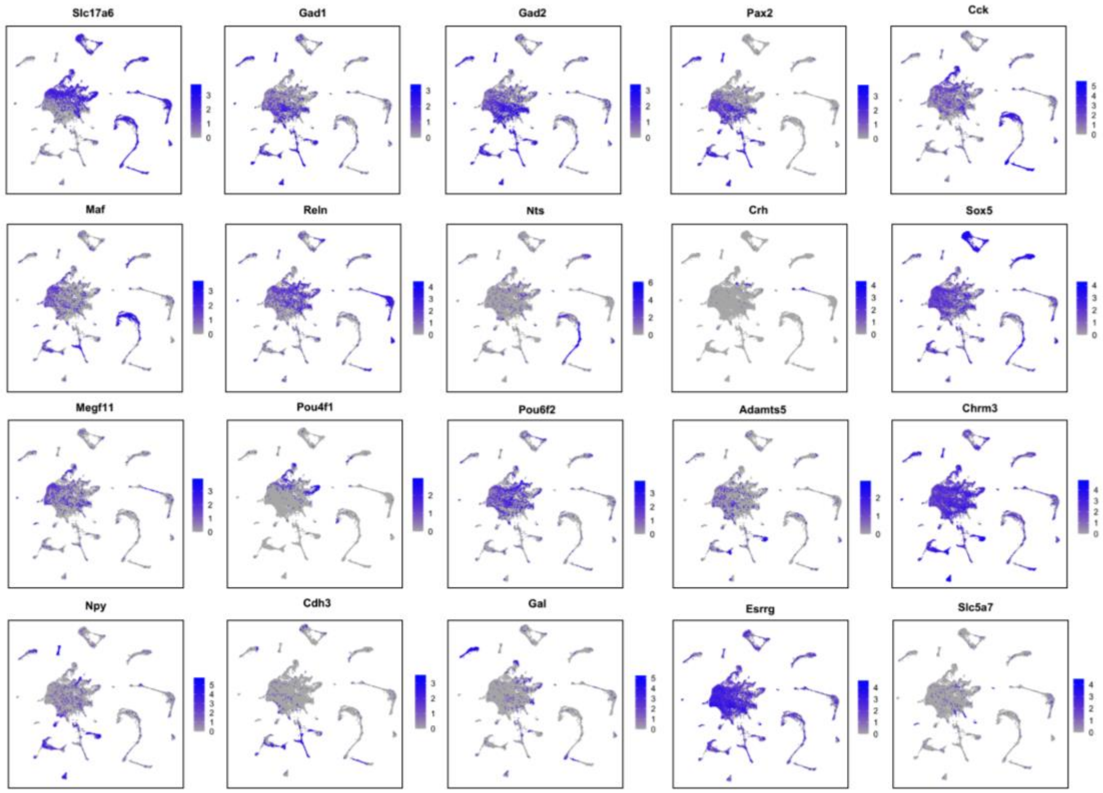

Supplementary Fig. 4. Markers of Neuronal Subpopulations. a. Featureplots showing markers for families of neurons. More can be visualized on https://seqseek.ninds.nih.gov/spinalcordinjury. **b.** Annotations of neurons using label transfer from the atlas of mouse lumbar spinal cord cell types, Russ et al. **c.** Subclustering of neurons and their annotations, highlighting a cluster that is not easily annotated using label transfer, but rather is defined by RAGs. **d.** Volcano plot showing differentially-expressed genes in the uninjured and injured RAG+ Cluster (cluster 23).

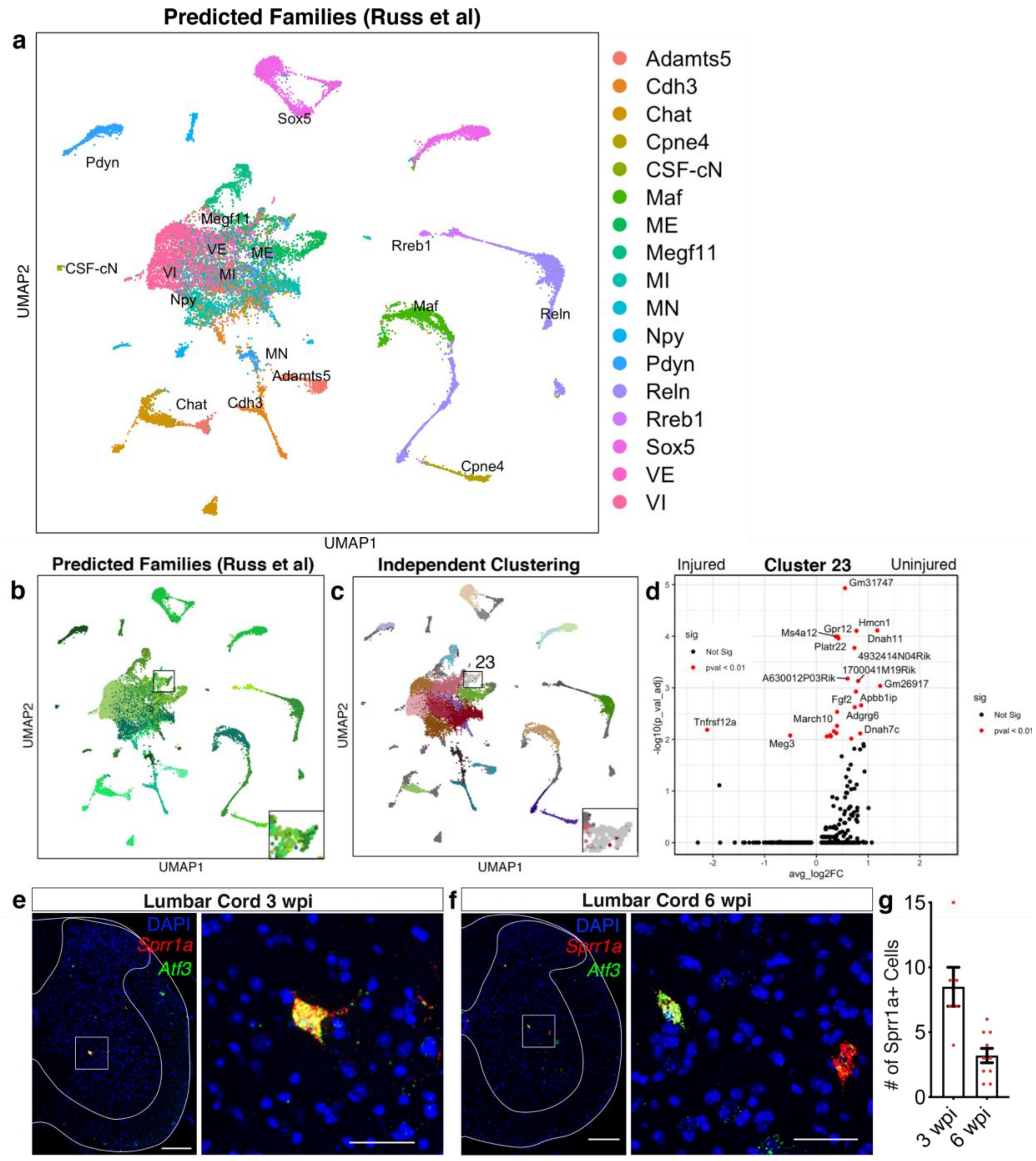

Supplementary Figure 5 a-b. UMAP of neurons classified by label transfer from Russ et al. 2021. **c.** UMAP of neurons clustered without label transfer, with cluster 23 expressing RAGs. **d.** Volcano plot of the differential gene expression after injury within cluster 23. The RAG *Tnfrsf12a* is the only significant gene upregulated after injury. Black dots indicate genes that were not significantly different. Red dots indicate significant genes, $p < 0.01$. Wilcox rank sum test. **e-f.** *In situ* hybridization of the RAGs *Sprr1a* and *Atf3* at (**d**) 3 wpi and (**e**) 6 wpi. Scale bars are 200 and 50 µm, respectively. **g.** Quantification of the number of *Sprr1a* in tissue at 3 and 6

wpi. Error bars indicate ± SEM; N = 6 and 10 animals, respectively. Source data are provided as a Source Data file.

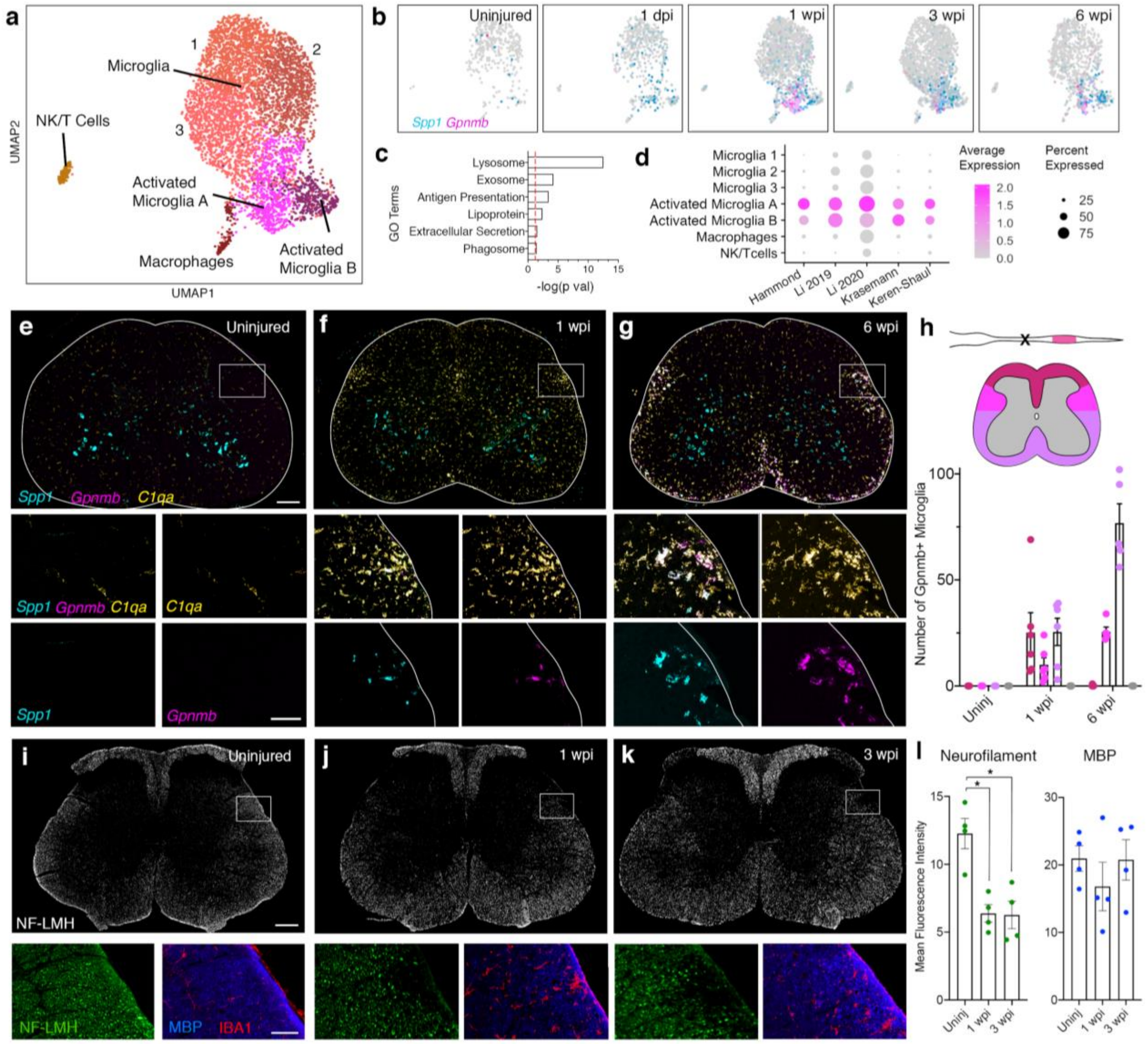

Supplementary Figure 6. Microglia Expansion and after Injury and the Emergence of a "Trauma Associated Microglia" a. UMAP of the 7 microglia subtypes. **b**. Featureplot of two marker genes of the "activated microglia A" population, *Spp1* (cyan) and *Gpnmb* (magenta), split by the timepoint (Uninjured, 1 dpi, 1 wpi, 3 wpi, and 6 wpi). **c**. GO analysis for "activated microglia A" marker genes. Red dotted line indicates -log(p-value) 1.3 (p value 0.05). P values (adjusted) were calculated using Benjamini-Hochberg false discovery rate (FDR). **d**. Dotplot comparing top 5 marker genes from previous studies of postnatal and disease-associated microglia. (*Clec7a*, not detected in our dataset, was excluded.) **e-g**. RNAscope *in situ* hybridization showing expression of *Spp1* (cyan) and *Gpnmb* (magenta) and *C1qa* (yellow) in uninjured, 1 wpi, and 6 wpi lumbar spinal cord. Scale bars are 200 µm in top row and 50 µm in middle and bottom rows. **h**. Number of Gpnmb+ microglia (quantified by *C1qa*, *Gpnmb* double-

positive cells) in each of the following regions in the spinal cord: dorsal funiculus (magenta), dorsal-lateral funiculus (pink), ventral funiculus (purple) and grey matter (grey). Error bars indicate mean ± SEM (N = 4 animals). **i-k**. Immunohistochemistry staining for MBP, IBA1 and Neurofilament light, medium and heavy (LMH). Scale bars are 200 µm in top row and 50 µm in bottom row. **l**. Quantification of fluorescence intensity of neurofilament-LMH and MBP in the region boxed i-k, in the dorsal-lateral white matter. * = p-val < 0.001, two-sided unpaired t-test. Error bars indicate mean ± SEM; N = 4 animals. Source data are provided as a Source Data file.

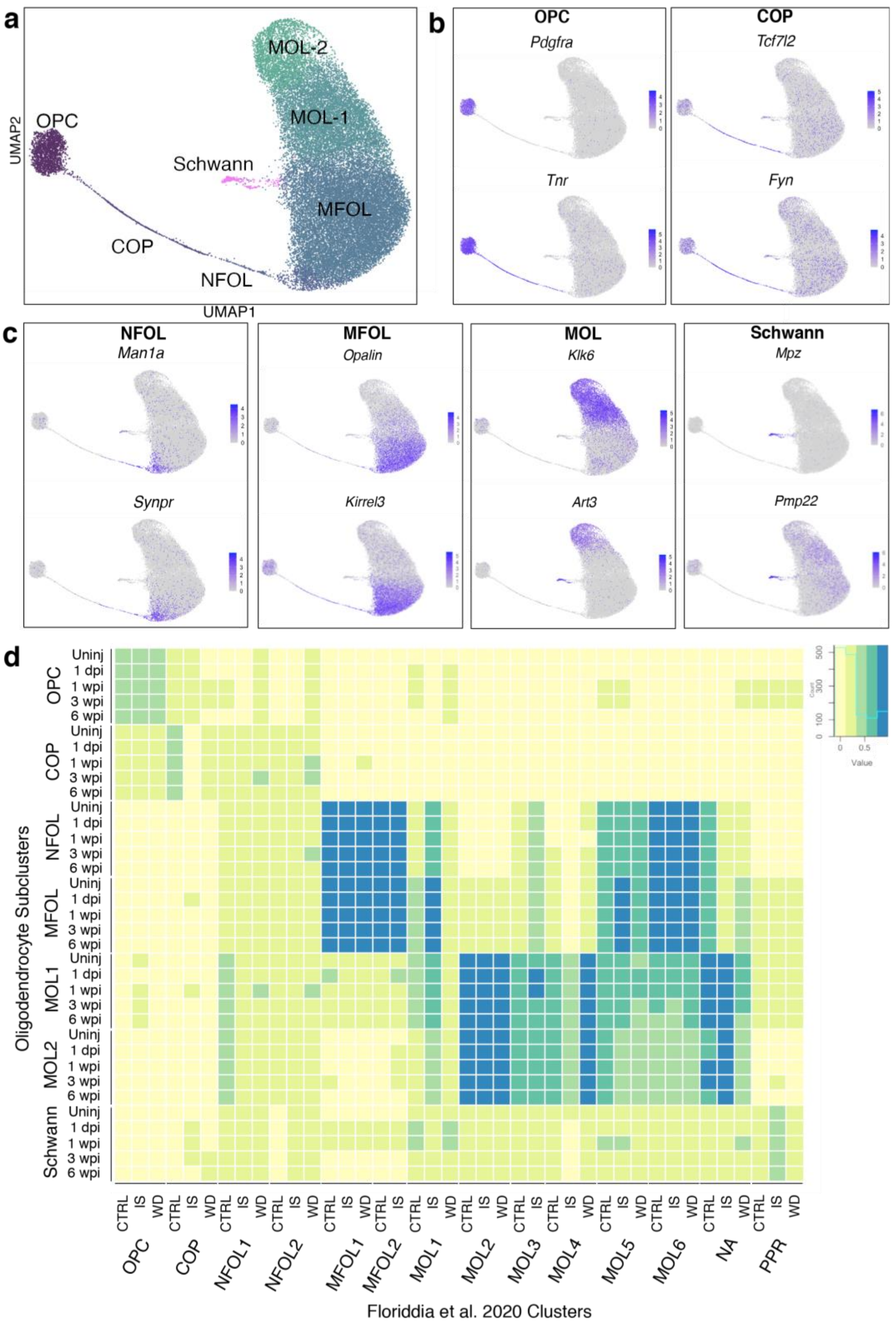

Supplementary Fig. 7. Oligodendrocyte Subclusters. a. UMAP of oligodendrocyte subclusters. **b-c.** Featureplots of genes differentiating oligodendrocyte subclusters. **d.** Pearsons correlation of top 2,000 variable genes between oligodendrocyte subclusters, split by injury condition

compared to oligodendrocyte subclusters from Floriddia et al. 2020, split by injury condition. Values are colored from 0 (yellow) to 1 (blue).

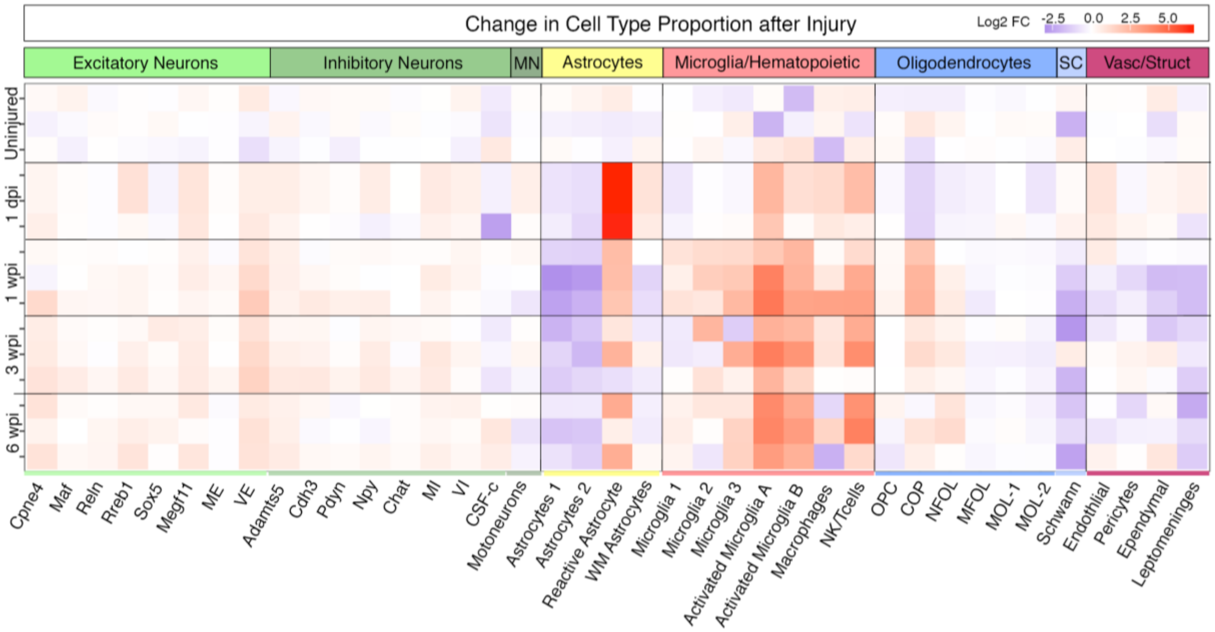

Supplementary Fig. 8. Changes in Cell Type Proportion after Injury. Heatmap showing the change in cell type proportion after spinal cord injury. Log2 fold change was calculated based on the average percent of a sample in the uninjured cord. Negative (decreases) in proportion are colored in blue and positive (increases) in proportion are colored in red (N = 3 animals per time point).

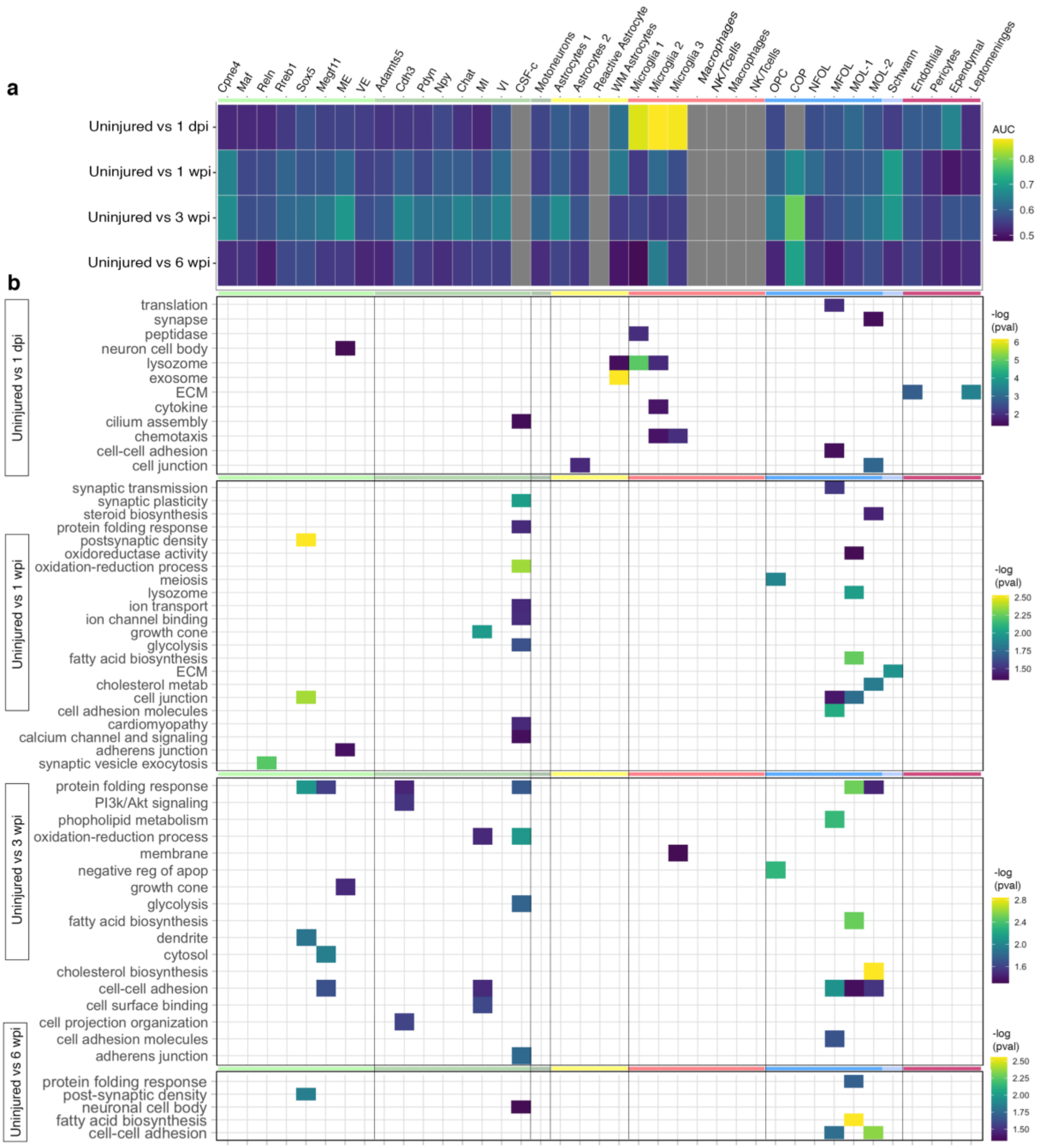

Supplementary Fig. 9. Cell Type Specific Changes in Gene Expression after Injury
a. Cell types ranked based on responsiveness by Augur. AUC score represented from dark blue to yellow. Clusters with insufficient number of cells in the uninjured timepoint are plotted in grey. **b**. Pathway analysis for differentially expressed genes between uninjured and injured timepoints. Tiles are colored by -log(p value) of GO and KEGG pathway clusters. -log(p-value) 1.3 = p value 0.05. P values (adjusted) were calculated using Benjamini-Hochberg false discovery rate (FDR). Source data are provided as a Source Data file.

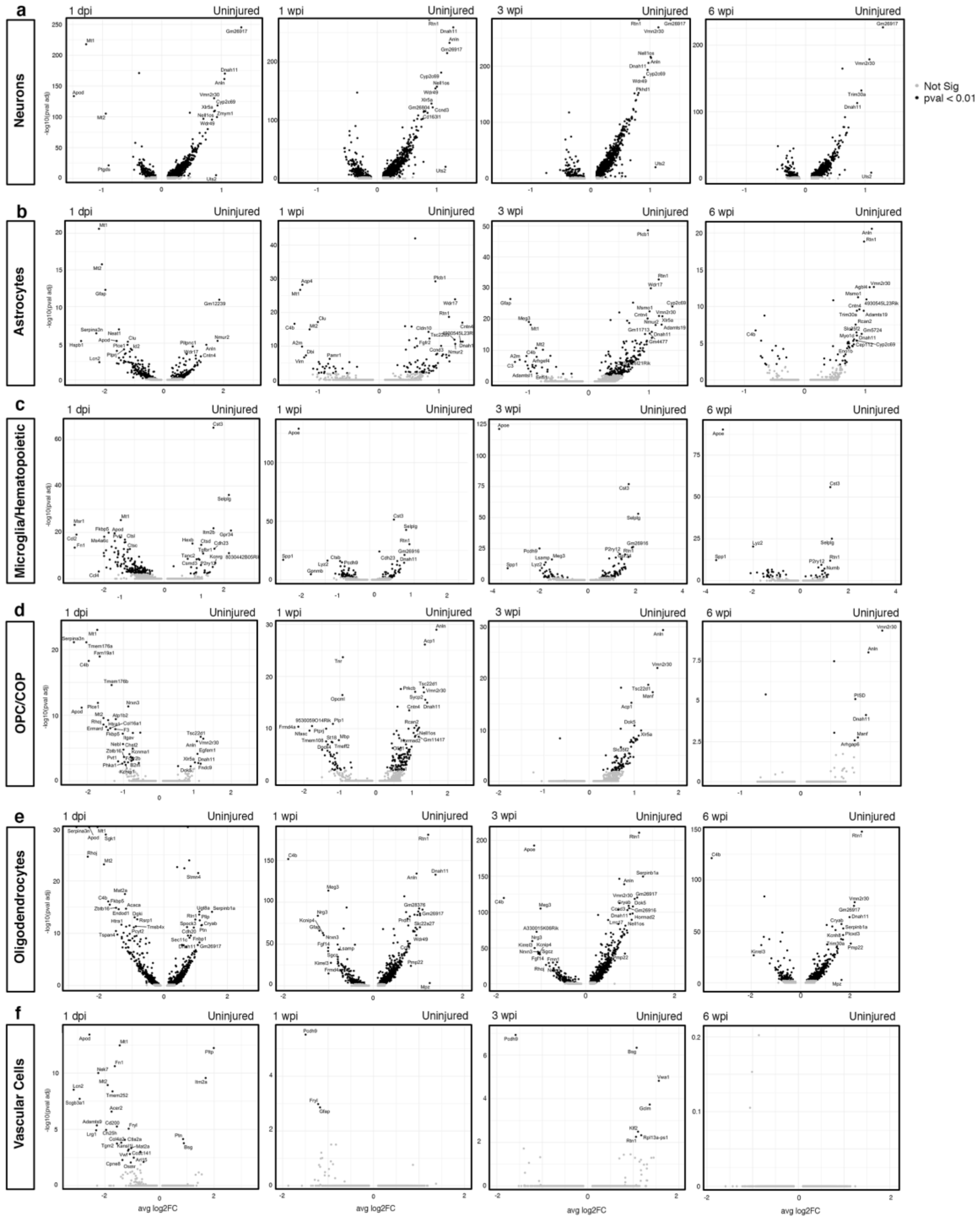

Supplementary Fig. 10. Differential Gene Expression in Cell Types after Injury. a-f. Volcano plots showing differential gene expression between **a.** neurons **b.** astrocytes **c.** microglia/hematopoietic cells **d.** oligodendrocyte progenitor cells (OPCs) and oligodendrocyte precursor cells (COPs). **e.** oligodendrocytes and **f.** vascular cells. Differential gene expression was calculated using Wilcox test. Significant genes are colored in black (p < 0.01) and not

significant genes are colored in grey. Genes with > 0.8 average log2 fold change are labeled by name. Source data are provided as a Source Data file.

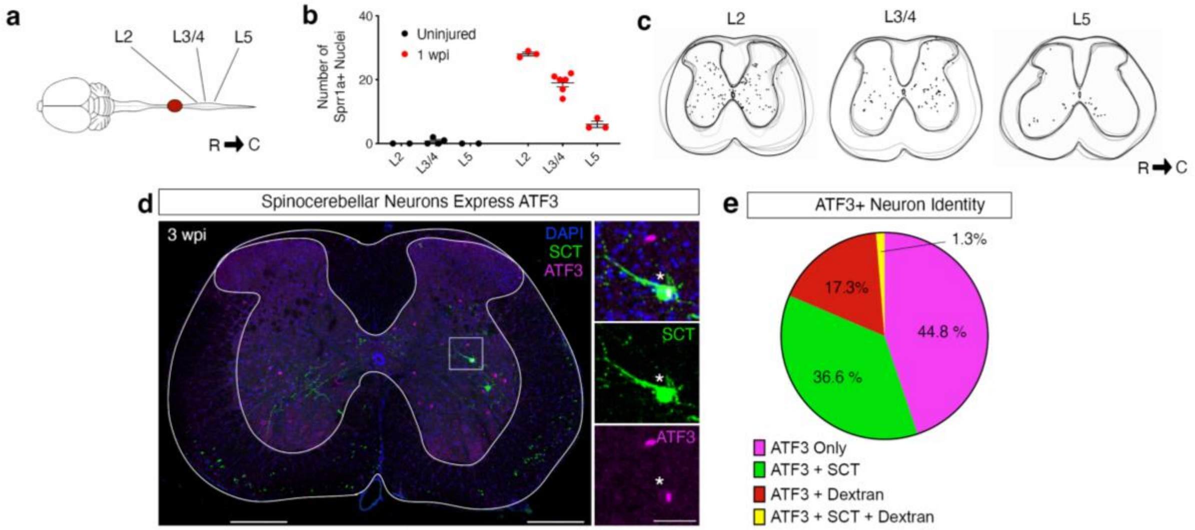

Supplementary Fig. 11. a. Schematic showing the rostral-caudal axis of L2, L3/4, and L5 in relation to the thoracic contusion injury. **b.** Quantification of *Sprr1a+* cells in the uninjured and 1 wpi lumbar cord along the rostral-caudal axis. **c.** Spatial summary of *Sprr1a* in the lumbar cord along the rostral-caudal axis across multiple animals (N = 4 animals). **d.** Immunostaining of ATF3, with stereotaxically-labeled spinocerebellar neurons. Representative replicate is shown. Scale bars are 200 and 50 µm, respectively. **e.** Quantification of ATF3+ neurons, coinciding with SCT and or dextran-labeling. (N = 4 animals.) Data from one independent cohort is shown. The results of this experiment were replicated in a second cohort.

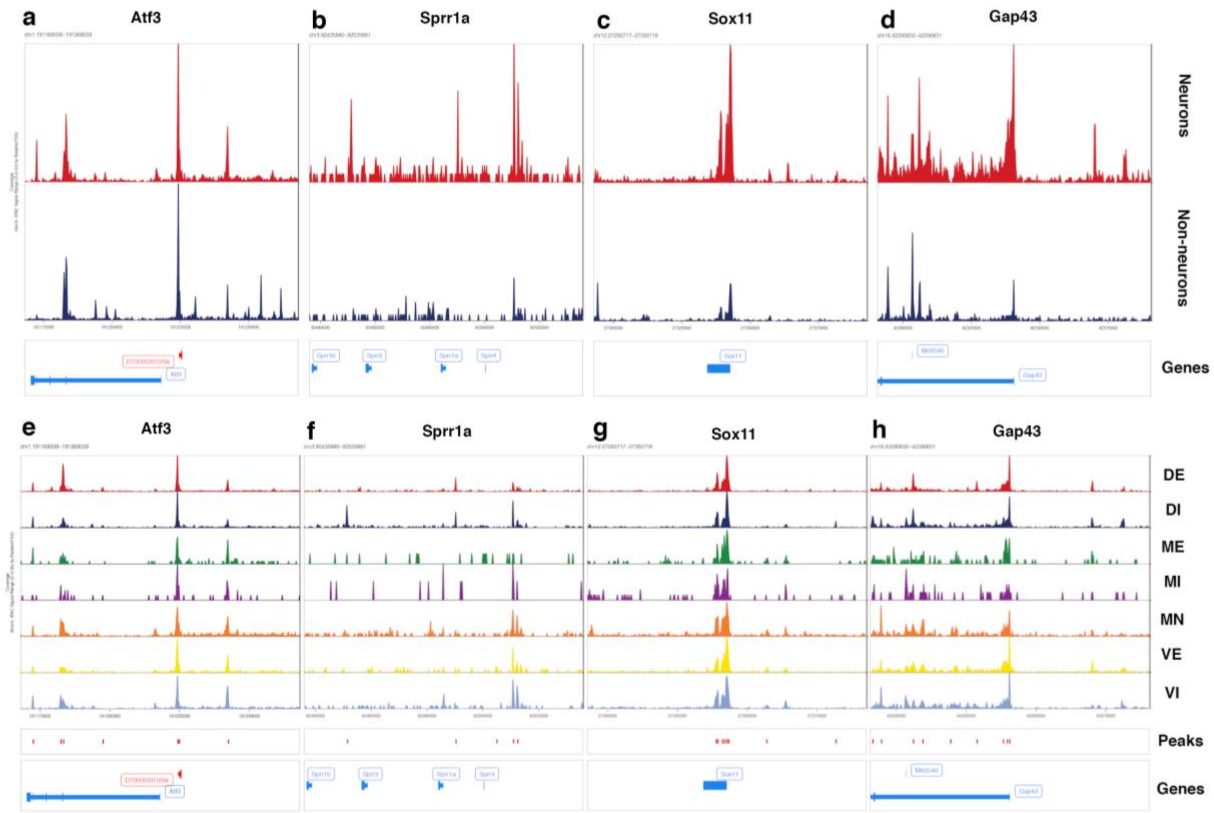

Supplementary Fig. 12. snATAC-seq to Examine Predisposition of RAG Expression in of Spinal Cord Cells. a-d. Plot tracks of open chromatin regions for RAGs in neurons and non-neurons. **e-h.** Plot tracks of open chromatin regions for RAGs across neuronal subtypes, including dorsal excitatory (DE), dorsal inhibitory (DI), mid-excitatory (ME), mid-inhibitory (MI), motoneurons (MN), ventral excitatory (VE), and ventral inhibitory (VI).

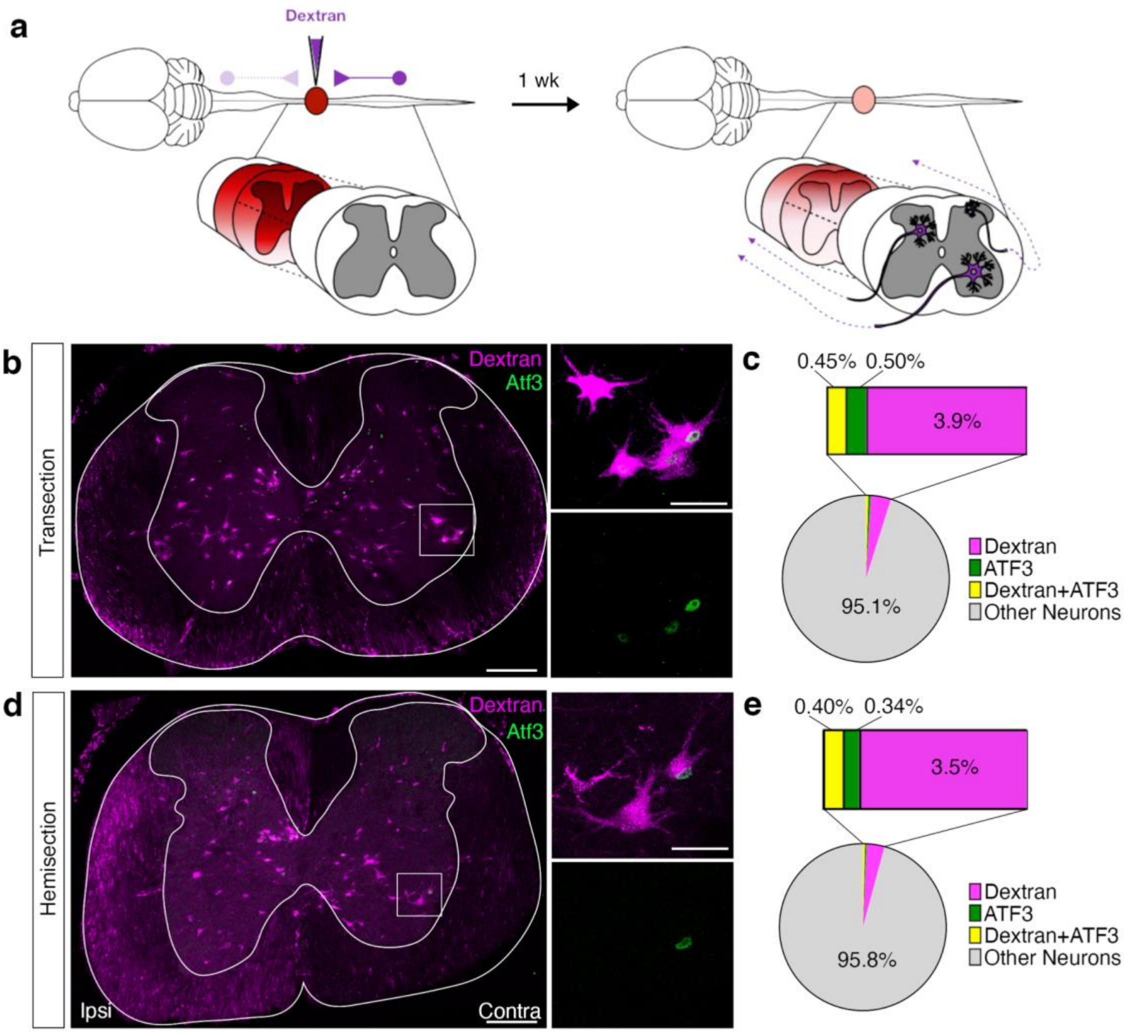

Supplementary Fig. 13. Dextran Labels Directly Injured Ascending Neurons, a Subset of ATF3+ Neurons. a. Schematic of dextran injection and injury. **b.** Immunohistochemistry of ATF3 on dextran-labeled thoracically transected lumbar spinal cord 1 wpi. Scale bars are 200 and 50 µm, respectively. **c.** Quantification of dextran and ATF3 as a proportion of all NeuN-expressing cells after thoracic transection. (N = 4 animals.) **d.** Immunohistochemistry of ATF3 on dextran-labeled thoracically lateral hemisectioned lumbar spinal cord 1 wpi. Scale bars are 200 and 50 µm, respectively. **e.** Quantification of dextran and ATF3 as a proportion of all NeuN-expressing cells after thoracic lateral hemisection. (N = 4 animals.)

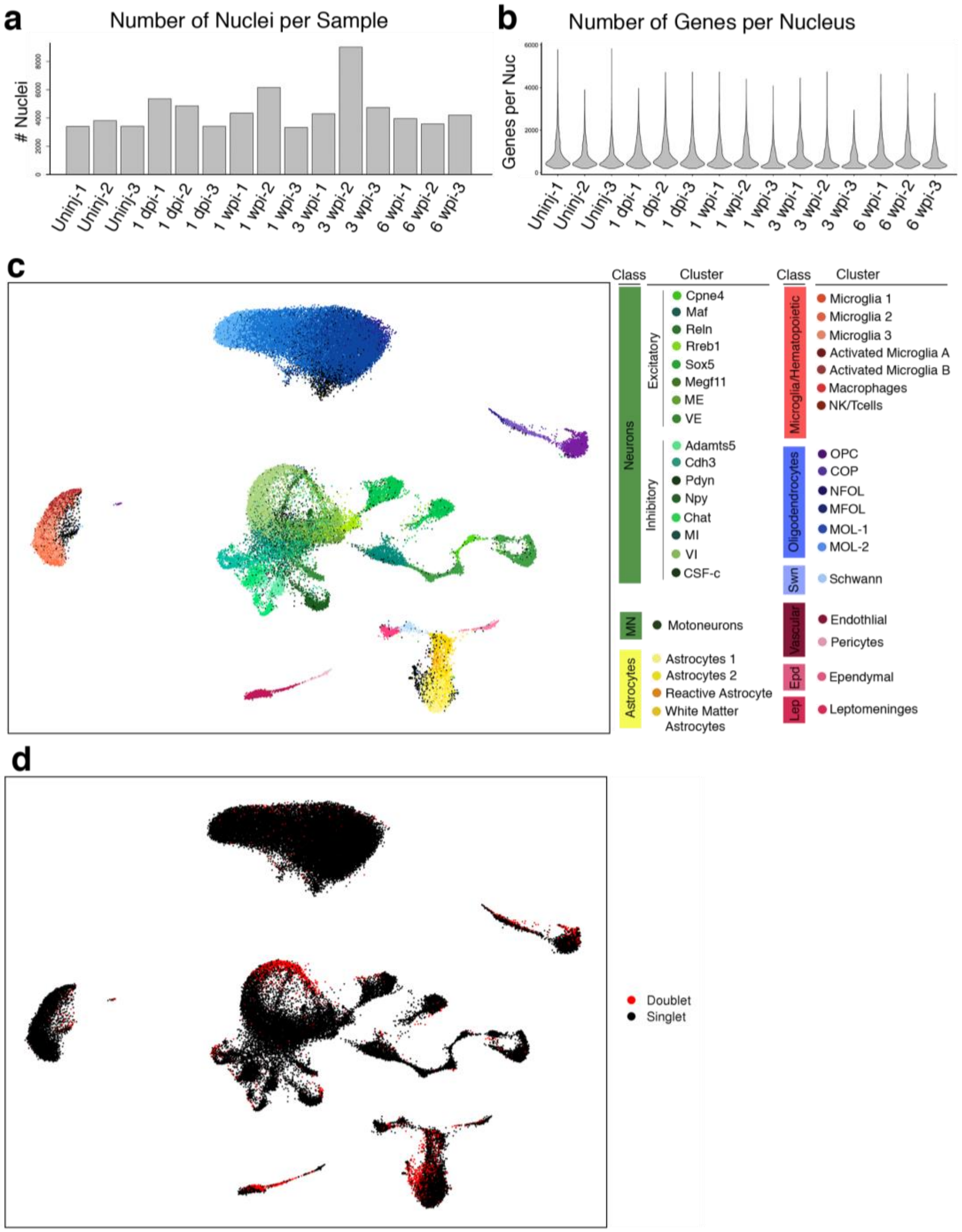

Supplementary Fig. 14. Quality Control and Doublet Identification. a. The number of nuclei per sample. **b.** The number of genes per nucleus in each sample. Source data are provided as a Source Data file. **c.** UMAP of all nuclei prior to removal of doublets. Doublets **d.** Identification of doublets using doublet finder.

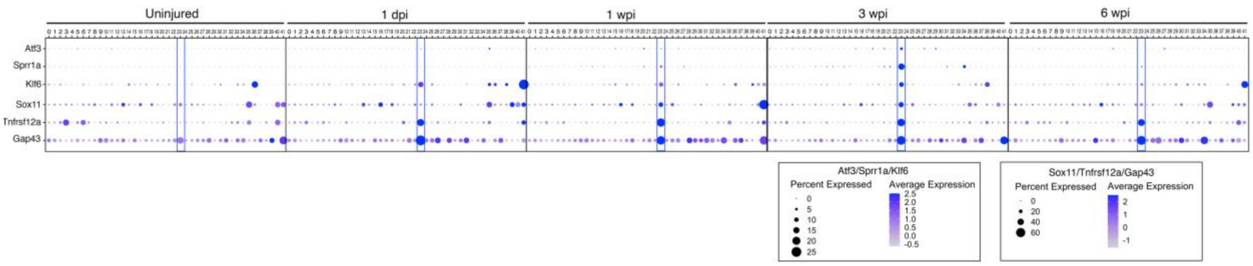

Supplementary Fig. 15. RAGs within Neuronal Subtypes Over Time. A dotplot showing the expression of the RAGs Atf3, Sprr1a, Klf6, Sox11, Tnfrsf12a and Gap43 in uninjured and injured timepoint. Average expression is indicated from grey (low) to blue (high).
