# Supplementary material for: Single cell atlas of spinal cord injury in mice reveals a pro-regenerative signature in spinocerebellar neurons
Source: Nat Commun. 2022 Sep 26;13:5628. doi: 10.1038/s41467-022-33184-1 (PMC9513082; doi:10.1038/s41467-022-33184-1)
Supplement: Supplementary file 2 — Description of Additional Supplementary Files [file 41467_2022_33184_MOESM2_ESM.pdf]

Description of Additional Supplementary Files

Supplementary Dataset 1: Kinematics of Mice after Injury Source Data. Limb movements were evaluated while running on a horizontal walkway. Bilateral leg kinematics were captured with the Vicon Motion Systems, UK (combining 12 infrared cameras) for tracking with reflective markers on the crest, hip, knee, ankle joints and distal toes. The limbs were modelled as an interconnected chain of segments. Based on these, a total of 80 gait parameters were computed for each limb for each gait cycle. Contains temporal gait features, limb trajectories, drag, stability, joint angles and segmental oscillations, velocities, intralimb temporal coupling and robotic support features.

Supplementary Dataset 2: Top 20 Markers for the 39 Clusters in Sequencing Dataset. For each of the 39 clusters, the top 20 markers are shown.

Supplementary Dataset 3: Proportion and Number of Nuclei for Replicates by Cell Type. For each cell type by sample, the proportion of a given sample and the number of nuclei are reported.

Supplementary Dataset 4: AUGUR Scores for Each Cluster. The AUGUR AUC (area under the curve) scores are reported for each cell type and timepoint. Source Data for Supplementary Figure 9a.

Supplementary Dataset 5: GO Pathway Analysis Source Data. Pathway analysis results for differentially expressed genes between uninjured and injured timepoints. Values are reported as -log(p value) of GO and KEGG pathway clusters. P values (adjusted) were calculated using Benjamin-Hockberg false discovery rate (FDR). Dataset is Source Data for Supplementary Figure 9b.

Supplementary Dataset 6: Differentially Expressed Genes Source Data. Differentially expressed genes, average log2 fold change (avg_log2FC) and adjusted p values (p_val_adj) calculated using the Wilcox test.

Supplementary Dataset 7: Quality Control Values. Contains the number of nuclei per sample before and after quality control filtering as well as the average number genes per nucleus for each sample.
