# Supplementary material for: Single cell atlas of spinal cord injury in mice reveals a pro-regenerative signature in spinocerebellar neurons
Source: Nat Commun. 2022 Sep 26;13:5628. doi: 10.1038/s41467-022-33184-1 (PMC9513082; doi:10.1038/s41467-022-33184-1)
Supplement: Supplementary file 10 — Reporting Summary [file 41467_2022_33184_MOESM10_ESM.pdf]

nature portfolio

Corresponding author(s): Ariel J Levine

Last updated by author(s): August 8, 2022

Reporting Summary

Nature Portfolio wishes to improve the reproducibility of the work that we publish. This form provides structure for consistency and transparency in reporting. For further information on Nature Portfolio policies, see our Editorial Policies and the Editorial Policy Checklist.

Statistics

For all statistical analyses, confirm that the following items are present in the figure legend, table legend, main text, or Methods section.

n/a	Confirmed	
☐	☒	The exact sample size (*n*) for each experimental group/condition, given as a discrete number and unit of measurement
☐	☒	A statement on whether measurements were taken from distinct samples or whether the same sample was measured repeatedly
☐	☒	The statistical test(s) used AND whether they are one- or two-sided<br>*Only common tests should be described solely by name; describe more complex techniques in the Methods section.*
☒	☐	A description of all covariates tested
☐	☒	A description of any assumptions or corrections, such as tests of normality and adjustment for multiple comparisons
☐	☒	A full description of the statistical parameters including central tendency (e.g. means) or other basic estimates (e.g. regression coefficient) AND variation (e.g. standard deviation) or associated estimates of uncertainty (e.g. confidence intervals)
☐	☒	For null hypothesis testing, the test statistic (e.g. *F*, *t*, *r*) with confidence intervals, effect sizes, degrees of freedom and *P* value noted<br>*Give P values as exact values whenever suitable.*
☒	☐	For Bayesian analysis, information on the choice of priors and Markov chain Monte Carlo settings
☒	☐	For hierarchical and complex designs, identification of the appropriate level for tests and full reporting of outcomes
☐	☒	Estimates of effect sizes (e.g. Cohen's *d*, Pearson's *r*), indicating how they were calculated

Our web collection on statistics for biologists contains articles on many of the points above.

Software and code

Policy information about availability of computer code

Data collection	Custom MATLAB-based code for quantification of cell counts is available at https://github.com/ArielLevineLabNINDS/CellCounter (doi: 10.5281/zendo.6967482). Custom python-based code for quantification of pixels on thresholded images is available at https://github.com/ijhua/pixel_counts. Code for Augur is available at https://github.com/neurorestore/Augur.
Data analysis	Analysis was performed in R (version 3.6.1), using the following packages: Seurat (version 3.2.2), RColorBrewer (version 1.1-2), ggplot2 (version 3.3.2), ggrepel (version 0.8.2), dbplyr (version 1.4.4), hrhrthemes (version 0.8.0), plyr (version 1.8.6), viridis (version 0.5.1), tibble (version 3.0.3), tidyverse (version 1.1.0), ArchR (version 1.0.1), and MACS2 (version 2.2.7.1). Statistical tests were performed in Prism (version 9.0.0). Code for Augur is available at https://github.com/neurorestore/Augur.

For manuscripts utilizing custom algorithms or software that are central to the research but not yet described in published literature, software must be made available to editors and reviewers. We strongly encourage code deposition in a community repository (e.g. GitHub). See the Nature Portfolio guidelines for submitting code & software for further information.

Data

Policy information about availability of data

All manuscripts must include a data availability statement. This statement should provide the following information, where applicable:

- Accession codes, unique identifiers, or web links for publicly available datasets
- A description of any restrictions on data availability
- For clinical datasets or third party data, please ensure that the statement adheres to our policy

A searchable version of this data is available at https://seqseek.ninds.nih.gov/spinalcordinjury. Raw sequencing data and count matrices have been deposited to the Gene Expression Omnibus (GSE172167, https://www.ncbi.nlm.nih.gov/geo/query/acc.cgi?acc=GSE172167). Source data are provided with this paper.

Human research participants

Policy information about studies involving human research participants and Sex and Gender in Research.

Reporting on sex and gender	No human participants were involved in this study.
Population characteristics	No human participants were involved in this study.
Recruitment	No human participants were involved in this study.
Ethics oversight	No human participants were involved in this study.

Note that full information on the approval of the study protocol must also be provided in the manuscript.

Field-specific reporting

Please select the one below that is the best fit for your research. If you are not sure, read the appropriate sections before making your selection.

[x] Life sciences [ ] Behavioural & social sciences [ ] Ecological, evolutionary & environmental sciences

For a reference copy of the document with all sections, see nature.com/documents/nr-reporting-summary-flat.pdf

Life sciences study design

All studies must disclose on these points even when the disclosure is negative.

Sample size	Sample sizes are estimated based on previous studies, with at least 4 animals per condition for anatomical and histological work, and 3 animals per condition for snRNAseq. For anatomical and histological work, 4 animals per condition enabled statistical analysis despite a small amount of variability expected after a spinal cord contusion injury. For snRNAseq, 3 animals were chosen as the minimum number to allow statistical analysis of each timepoint, while balancing sequencing costs.
Data exclusions	Spinal Cord Injury: Two days after injury, all mice were evaluated in an open field, and all animals exhibiting any hindlimb movements were not further studied. For single nucleus RNA sequencing experiments, a larger cohort of mice was taken through kinematic analysis, and 3 mice representative of each timepoint were selected. For histology experiments, at least 4 mice were used for each condition. Mice with bone-hit contusions or injuries that fell a standard deviation outside of the average behavior for each timepoint were excluded. Due to animal care requirements injury experiments were not performed blinded.<br>Viral Labeling: Mice were excluded when viral labeling showed less than 20 cells in the lumbar spinal cord.
Replication	AAV-injections were replicated in two completely independent experimental cohorts. Mice were randomly assigned to injury groups and AAV-injection groups in each cohort, with AAV injections occurring two weeks prior to the injury surgery. Both cohorts were included in the final analysis.
Randomization	Animals were randomly assigned to injury groups or to AAV-injection groups.
Blinding	Due to animal care requirements injury experiments were not performed blinded. Quantification of immunohistochemistry was performed blinded.

Reporting for specific materials, systems and methods

We require information from authors about some types of materials, experimental systems and methods used in many studies. Here, indicate whether each material, system or method listed is relevant to your study. If you are not sure if a list item applies to your research, read the appropriate section before selecting a response.

Materials & experimental systems

n/a	Involved in the study
☐	☒ Antibodies
☒	☐ Eukaryotic cell lines
☒	☐ Palaeontology and archaeology
☐	☒ Animals and other organisms
☒	☐ Clinical data
☒	☐ Dual use research of concern

Methods

n/a	Involved in the study
☒	☐ ChIP-seq
☒	☐ Flow cytometry
☒	☐ MRI-based neuroimaging

Antibodies

Antibodies used

Primary antibodies:
IBA1 (dilution 1:100, Cedarlane Labs, 234006(SY)), TMEM 119 (dilution 1:100, Cedarlane Labs, 400004(SY)), CD11c (dilution 1:100, GeneTex, GTX74935), Myelin-MBP (dilution 1:100, BioLegend, 808402), NF-L (dilution 1:200, Cell Signaling, 2835S), NF-M (dilution 1:200, Cell Signaling, 2838S), NF-H (dilution 1:200, Cell Signaling, 2836S), NeuN (dilution 1:500, Millipore Sigma, ABN90P), CD68 (dilution 1:100, Abcam, ab125212), CNPase (dilution 1:200, Millipore Sigma, MAB326), GFAP (dilution 1:500, Agilent/Dako, Z033429-2), Cleaved Caspase 3 (dilution 1:100, Cell Signalling Tech, 9661L), and Phospho-IGF1R (dilution 1:100, Invitrogen, PA5-104773).

Secondary antibodies:
DAPI (dilution 1:1000, Thermo Fisher Scientific, 62248), Goat anti-Hamster IgG Alexa Fluor 647 (dilution 1:200, Jackson ImmunoResearch, 127-605-160), Goat anti-Rabbit IgG Alexa Fluor 430 (dilution 1:200, Thermo Fisher Scientific, A11064), Goat anti-Rabbit IgG Alexa Fluor 430 (dilution 1:200, Thermo Fisher Scientific, A11064), Goat anti-Rabbit IgG Alexa Fluor 594 (dilution 1:200, Thermo Fisher Scientific, A11037), Goat anti-Mouse IgG1 Alexa Fluor 488 (dilution 1:200, Thermo Fisher Scientific, A21121), Goat anti-Mouse IgG1 Alexa Fluor 647 (dilution 1:200, Thermo Fisher Scientific, A21240), Goat anti-Mouse IgG2a Alexa Fluor 546 (dilution 1:200, Thermo Fisher Scientific, A21133), Goat anti-Mouse IgG2b Alexa Fluor 647 (dilution 1:200, Thermo Fisher Scientific, A21242), Goat anti-Guinea Pig IgG Alexa Fluor 546 (dilution 1:200, Thermo Fisher Scientific, A11074), Goat anti-Guinea Pig IgG Alexa Fluor 594 (dilution 1:200, Thermo Fisher Scientific, A11076), Donkey anti-Chicken IgG IRDye 680LT (dilution 1:200, Li-Cor Biosciences, 926-68028), Donkey anti-Rabbit IgG IRDye 800CW (dilution 1:200, Li-Cor Biosciences, 926-32213), Donkey anti-Chicken IgG Alexa Fluor 488 (dilution 1:500, Jackson ImmuoResearch, 703-545-155), and Donkey anti-Guinea Pig IgG Alexa Fluor 647 (dilution 1:500, Jackson ImmuoResearch, 706-605-148).

Validation

A combination of morphological criteria, spatial distribution, and co-staining with other markers was used to validate each antibody used. For IBA1, TMEM119, Myelin-MBP, NF-L, NF-M, NF-H, NeuN, CNPase, GFAP and DAPI, previous work from the lab and example images from the manufacturer's website were used to inform the validity of antibody staining. Furthermore, overlapping signal from NF-L, NF-M, NF-H and NeuN helped to validate the antibodies as neuronal-specific. Similarly, overlapping and non-overlapping signal from IBA1 and TMEM119 helped to validate the antibodies as microglia and monocyte or microglia-specific. CD11c, CD68, Cleaved Caspase 3 and Phospho-IGF1R were validated by comparison to manufacturer's descriptions and example images.

Animals and other research organisms

Policy information about studies involving animals; ARRIVE guidelines recommended for reporting animal research, and Sex and Gender in Research

Laboratory animals

Mice for RNA sequencing were female C57BL/6 (12-30 weeks of age). For all other experiments, balanced samples of male and female C57BL/6 mice (12-30 weeks of age) were used. Mice used in this study were housed under a 12-h light–dark cycle (06:00–18:00 light), with ad libitum access to food and water. Room temperature was between 20–26 °C and humidity was between 30–70%.

Wild animals

No wild animals were used in this study.

Reporting on sex

Mice for RNA sequencing were female. All other experiments used balanced samples of male and female mice.

Field-collected samples

This study did not involve samples collected from the field.

Ethics oversight

All procedures and experiments were approved by the Veterinary Office of the Canton of Geneva (Switzerland) and the National Institute of Neurological Disorders and Stroke Animal Care and Use Committee (NINDS protocol #1384).

Note that full information on the approval of the study protocol must also be provided in the manuscript.
